# The Aging of Polymers under Electromagnetic Radiation

**DOI:** 10.3390/polym16050689

**Published:** 2024-03-03

**Authors:** Chrysanthos Maraveas, Ioannis Vasileios Kyrtopoulos, Konstantinos G. Arvanitis, Thomas Bartzanas

**Affiliations:** Department of Natural Resources Development and Agricultural Engineering, Agricultural University of Athens, 75 Iera Odos Street, 11855 Athens, Greece; kyrtopoulos@aua.gr (I.V.K.); karvan@aua.gr (K.G.A.); t.bartzanas@aua.gr (T.B.)

**Keywords:** polymer, degradation, aging, ultraviolet radiation, composites

## Abstract

Polymeric materials degrade as they react with environmental conditions such as temperature, light, and humidity. Electromagnetic radiation from the Sun’s ultraviolet rays weakens the mechanical properties of polymers, causing them to degrade. This study examined the phenomenon of polymer aging due to exposure to ultraviolet radiation. The study examined three specific objectives, including the key theories explaining ultraviolet (UV) radiation’s impact on polymer decomposition, the underlying testing procedures for determining the aging properties of polymeric materials, and appraising the current technical methods for enhancing the UV resistance of polymers. The study utilized a literature review methodology to understand the aging effect of electromagnetic radiation on polymers. Thus, the study concluded that using additives and UV absorbers on polymers and polymer composites can elongate the lifespan of polymers by shielding them from the aging effects of UV radiation. The findings from the study suggest that thermal conditions contribute to polymer degradation by breaking down their physical and chemical bonds. Thermal oxidative environments accelerate aging due to the presence of UV radiation and temperatures that foster a quicker degradation of plastics.

## 1. Introduction

Polymers are long-chain and giant molecules obtained from diverse smaller molecules known as monomers. Polymers are, therefore, composed of multiple repeating monomers in extended chains that can, at times, be branched or cross-linked [1]. White [2] explains that polymer aging refers to its change of chemical properties over time. Such properties that change include toughness and strength, density, and reactivity towards aggressive chemical substances [2]. Polymer aging can be attributed to either a physical change or chemical alterations when curing a thermoset. Exposing polymers to thermal conditioning fosters the deterioration of their chemical properties due to temperature increase [2]. The aging process of polymers when exposed to ultraviolet (UV) rays has been subject to extensive research interest. UV radiation is defined as a typology of non-ionizing radiation, which the Sun and other artificial sources like tanning beds emit [2]. For instance, Yousif and Haddad [3] mention that exposing polymers to UV radiation leads to photooxidative degradation, which is akin to breaking their chains, generating radicals, and lowering their molecular weight. This process results in the mechanical properties of the polymer deteriorating and waste generation after some unpredictable duration [3]. Therefore, exposing polymers to UV radiation enhances their aging by hastening the loss of their chemical as well as physical properties.

Nearly all the procedures of accelerated polymer aging use radiations generated by different lamps, like xenon, metal halides, fluorescent lighting tubes, and mercury, which help catalyze the process [4]. In agreement with this perspective, Frigione and Rodríguez-Prieto [5] argue that accelerated aging is possible as photo-chemical processes principally facilitate polymers’ degradation. In alignment, Pickett et al. [6] suggest that polymer aging degrading mechanisms occur when the irradiated plastic material relies on irradiation wavelengths, radiative energy, and its chemical structure. It is worth mentioning that the Earth receives UV radiations of the wavelength range 290–400 nm [4]. However, Frigione and Rodríguez-Prieto [5] suggest that devices capable of reproducing the Sun’s natural exposure on a polymer’s surface should use wavelength thresholds of 290–320 nm. These wavelengths are important to consider when deciding which range to employ to attain the best results for polymer aging.

**Figure 1:**
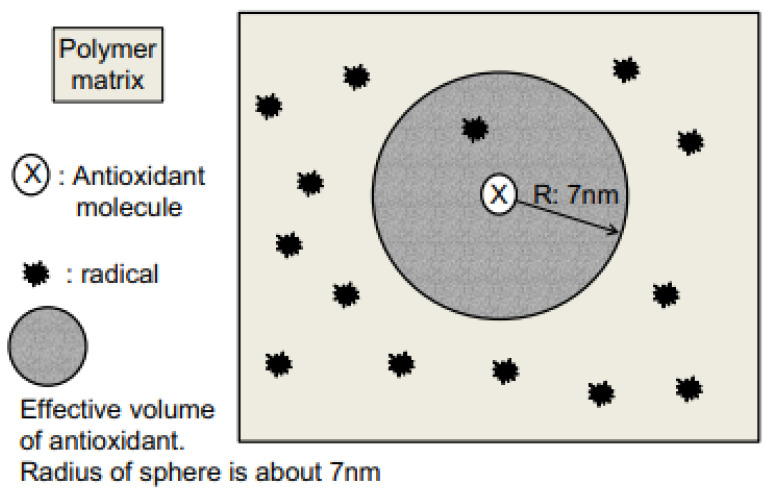
The interaction of an antioxidant with a polymer [8].

One such thermal method for using UV radiation to foster a polymer’s aging is chemiluminescence. This method, which is reported to have been in operation since the 1960s, entails using either photooxidation or thermal oxidation to emit weak light that is used to promote the aging of a polymer [7]. Kockott [7] suggests that a luminescent reaction terminates two peroxy radicals, leading to light emission by the excited carbonyl group when regaining its natural state. This process is especially prevalent in polyolefins’ aging. In contrast to using thermal radiation to achieve the aging of a polymer, antioxidation has been suggested by Seguchi et al. [8] as the most suitable technique for polymer stabilization. The authors explain that antioxidation is useful in stabilizing most polymer materials, which reduces polymer degradation. Figure 1 below shows a model for an antioxidative reaction with a polymer in a thermal environment.

In Figure 1, the antioxidant depresses the production of radicals in the polymer matrix and effectively delocalizes energy for thermal activation. In this regard, a small proportion of an antioxidant is mixed with a polymer to stabilize it. Antioxidants help extend the lifetime of polymer materials, with diverse typologies adapted for use in different environments. In contrast, Seguchi et al. [8] contend that using oxidation schema can help create a chain reaction through the peroxy-radical as well as hydro-peroxide. In this mechanism, the polymers’ antioxidant terminates free radicals and causes the hydro-peroxides of polymer chains to decompose as they undergo oxidation. However, using UV radiation to foster polymer aging can be dangerous as small particles are deposited into the surrounding ecosystems, leading to environmental degradation. This issue is prevalent since aged plastics have to be replaced, which leads to plastic pollution. For instance, according to Liu et al. [9], plastic debris exposed to UV radiation deforms and creates microplastics <5 mm in size. These particles can enter the surroundings by use of personal care products and plastic fabrication [9]. Therefore, aged plastic materials contribute to the degradation of the environment.

In terms of testing procedures, the lifespan of polymer materials and coatings increases over time, meaning elongated testing periods. However, much longer testing periods are unacceptable due to economic reasons [10,11]. In this regard, economic constraints have placed testing techniques under extreme pressure in a bid to reduce the durations for testing [12]. As such, Kockott [7] has suggested applying analytical methods in characterizing polymers’ aging processes at molecular levels. Kockott [7] asserts that qualitatively altering the polymer at the macroscopic level is based on changing its molecular material level. As such, Kockott [7] suggests the need to identify analytical methods’ molecular changes to enhance the determination of qualitative alterations before their detection at the macroscopic level. Essentially, this suggestion implies that testing periods can be shortened through analytical methods compared to measuring qualitative changes at the microscopic level. Polymer aging can also be accelerated by applying UV-A and UV-B lamps in laboratory tests. For instance, according to Fiorio et al. [13], UV-A aging contributes to a severe degradation of a polymer’s irradiated surface (<100 μm), coupled with physical aging inducement. The surfaces exposed to UV-A get embrittled and visibly degrade, which influences the deterioration of ABS’s mechanical properties [13,14]. Similarly, a study by Ma et al. [15], which examined the aging effect of UVB on concrete polymers, established that extreme changes in temperature and UV radiation led to the deterioration of the concrete’s flexural performance as the aging time advances. Precisely, the findings indicate that for an equivalent aging period of four years, the polymer concrete deteriorated in terms of its flexural strength by 8.4% [15]. Hence, these radiations accelerate the aging process of polymers.

Examining polymer durability requires relatively shorter timeframes of about three years compared to their duration in service [15]. Thus, studies of polymer durability have attempted to accelerate the aging by raising their aging temperatures during experimentation. This feature is integral in ensuring that a polymer’s loss of mechanical properties can be discerned within the duration of aging. After that, those measuring polymer durability are required to extrapolate the results from the extreme temperatures to service temperatures through the use of either empirical approaches or physical models [15]. However, the quality of such an extrapolation relies on manifold parameters like the number of degradation processes used, the potential couplings adopted, the homogeneity or heterogeneity of the degradation processes’ character, and the range of temperatures used to accelerate aging. Keeping aging temperatures extremely high can influence different degradation processes, discernible from those in service [15,16]. In this regard, there is a trade-off between the used temperature range and aging times seen in polymer durability investigations. Baklan et al. [17] assert that the rate and grade of photodegradation rely on different factors like a polymer’s chemical composition, the type of additives used, surface structure, and the prevalence of ultraviolet-sensitive molecules’ functional groups. For instance, polymers have different chemical compositions, including hydroxyl groups, oxygen-containing elements, and carbonyl, which can influence their degradation rates and grades [16]. Concerning the types of additives, Baklan et al. [17] explain that photoactive additives like titanium oxide foster polymer degradation, while light stabilizers like HALS safeguard the polymer from UV radiation. As such, different mechanisms can be used to prolong the durability of polymers by shielding them from the adverse impact of UV radiation.

However, anti-aging components can be used to shield polymers from degradation. For instance, Moraczewski et al. [18] examined the possibility of modifying polycaprolactone using plant extracts to enhance its aging resistance. The findings suggest that natural extracts from coffee, cocoa, and cinnamon could be used to provide anti-aging components for biodegradable polymers [18]. The aging experimentation of these polymeric materials is usually conducted at elevated temperatures, coupled with high relative humidity as well as consistent exposure to ultraviolet radiation for extensive hours [18]. This argument concurs with the perspective of Kockott [10], who indicated that polymeric materials like plastics, varnishes, and textiles alter their chemical and physical properties based on the parameters to which they are exposed. Two crucial exposure parameters for the aging process of polymers include incidental solar radiation and the object’s temperature [10]. As such, humidity and pollution from air and rain are not considered major parameters influencing the aging of polymers. Polymeric objects exposed to UV radiation absorb solar radiation, leading to the generation of free radicals, which initiates the degradation process and warming of the object relative to ambient temperatures [10]. Rodriguez et al. [19] concur with this assertion by arguing that as UV radiation interacts with polyolefins, it leads to photooxidative embrittlement, which is akin to random chain scission and cross-linking, and this ultimately influences a decline in the plastic’s average molecular weight. These impacts suggest that polymeric materials degrade over time as they are used due to their interactions with UV radiation.

With increased applications of polymers in real-world scenarios, understanding the conditions that facilitate their aging and degradation is crucial in shaping their practical use. The aging and degradation of polymers have real-world implications in areas where they are applied, such as agricultural plastics. For instance, Cosnita et al. [20] suggest that it is crucial to understand waste polymer composites’ stability vis-à-vis UV radiations, as there is a persistent increase in their outdoor applications. As these composite materials are prevalent in building materials, pavements, railway wall covers, carpeting railway crossings, and speed limiters’ panel streets, understanding their resistance to UV radiation is important for practitioners since their context of use exposes them to solar radiation [20]. Ghosh et al. [21] underscore that there is a dearth of published literature regarding the aging of all waste composites on the basis of wood and tire rubber. These waste products are available in abundance, and they are non-biodegradable [21]. Therefore, converting them into value-added products presents the need for sustainable development to reduce the inherent environmental burden.

Exposing polymer composites to hydrothermal environments contributes to their faster degradation and inhibits their performance. Indeed, Qi et al. [22] report that polymer composites reinforced with carbon fiber undergo oxidation, softening and hydrolysis when exposed to hydrothermal environments, leading to reduced stiffness as well as strength. Furthermore, Qi et al. [22] suggest that exposing such polymer composites to hydrothermal settings accelerates the interfacial delamination between their fiber and matrix. This phenomenon induces the failure of the interface and the ultimate damage of the composite [22]. Zhumadilova et al. [23] agree with the latter assertion, claiming that the color and execution features of liquid thermal insulation coatings are influenced by the combined impacts of diverse climatic conditions. Such conditions include solar radiation, as well as changes in temperature, moisture, and precipitation, among others [23,24]. In this regard, understanding the thermal performance of polymer composites is integral in conceptualizing their life and reliability and enhancing their application in construction and civil engineering fields.

Short-term aging is capable of altering the mechanical properties and morphological elements of polymers, especially those modified through bitumen, recycled plastic materials, and other wastes. This perspective is highlighted by Celauro et al. [25], who argue that bitumen that has been modified with polymers to offer enhanced performance is affected by the short-term degradation of polymer composites resulting from the high processing temperatures to which they are exposed. The same scenario occurs when modified binders are produced as the polymeric material is dispersed under extreme heat in the mass of the bitumen [25]. In concurrence, Zhu et al. [26] conducted a study to determine the impact of the polymer structure on modified binders’ physicochemical features and performance-related properties. The study also considered the influence of polymer content as well as aging conditions on the modified asphalt binders’ performance [26]. Through a polynomial experimental design, the study established that binder properties are impacted by both the polymer structure and the interaction between polymer content, structure, and aging [26]. For instance, polymer structure is integral in predominantly influencing the morphology of polymer particles in modified binders and fostering the stability of their storage [26]. Polymer content affects the polymeric feature peaks in the spectrum of infrared, elasticity, and elastic recovery, as well as the binders’ non-recoverable creep compliance. The aging condition of polymeric materials influences their variations in the oxidization of binders and their complex shear modulus [26,27]. As such, the polymer’s structural impacts are limited when the polymer content is low, while high polymer content substantially influences observable property differences.

Conducting this study is important to promote a nuanced understanding of the effects of electromagnetic radiation, both visible and infrared radiation, on polymers. Brandt et al. [27] suggest that polymer aging is influenced by three main factors, including UV radiation, extreme temperatures, and high humidity, which can be taken into account in isolation. This perspective implies that each of these elements can independently influence polymer aging since the outcomes of exposing polymeric materials to environmental conditions are mutually exclusive of the other conditions. Celina et al. [28] support the notion above by suggesting that exposing polymeric materials to combined-radiation hot environments accelerates their aging and degradation. Combined conditions of high-temperature radiations and oxidization are more intrinsically convoluted than conventional thermal degradation [28,29,30]. Understanding this phenomenon is of academic and practical relevance. For instance, it is crucial to understand polymer composites’ aging mechanisms in order to use them in thermal aging ecosystems or thaw environments [31,32]. Polymer composites have different mechanical behaviors that rely on the interface’s ability to move stress from the matrix to the fiber intended for reinforcement [32]. For this reason, determining the aging process of polymers under UV radiation can provide practical insights that can improve how testing procedures are implemented in routine contexts. Other researchers can also use the findings of this study to enhance their understanding of the effects of sunlight on the aging process of polymers and identify potential literature gaps on which to focus their further inquiries. The findings from this review are useful in enriching the current understanding of the aging process of polymers and provide future research directions.

The purpose of this paper is to review the literature on the effect of using electromagnetic radiation to foster the aging of polymers. To the best of the researcher’s knowledge, this is the first review on the topic, and is thus of novel research interest. The specific objectives of this study include:

- To explore the key theoretical positions on the range of UV radiation that affects the polymers and the radiation that generates heat for polymer decomposition;
- To review the testing procedures used for polymer aging evaluation and real-world applications that are impacted by the aging of polymers, especially agricultural plastics;
- To appraise the available technical methods for improving the performance of polymers under UV radiation and other radiation mediums to identify whether there are any promising technologies for meeting this need.

The rest of the paper is organized into the methodology, results, discussion, and conclusion sections. The methodology section details and justifies the search strategy and data extraction protocols used in this review. After that, the findings are presented by synthesizing the selected articles, followed by a discussion of the findings. Lastly, the conclusion section summarizes the research findings and highlights recommendations for practice and future research.

## 2. Materials and Methods

### 2.1. Research Philosophy

This study used an interpretivism research paradigm to explore the aging effect of polymers under radiation. Ryan [33] explains that the interpretivist research paradigm posits that truth and reality are culturally and historically situated, reflecting the subjective experiences of people and the meanings they attach to them. Similarly, Žukauskas et al. [34] contend that interpretivism entails developing knowledge subjectively based on people’s multiple perspectives, realities, and experiences. In this regard, the researcher deemed an interpretivism research paradigm as suitable for this literature review study to promote the subjective exploration of the aging effect of polymers when exposed to UV radiation. Drawing on and reviewing diverse perspectives from various authors was therefore important in understanding how they conceptualize the aging effect of polymers exposed to UV radiation.

### 2.2. Research Approach

The study used an inductive research approach to explore the aging effect of polymers under radiation. Inductive research involves the use of specific observations to help guide the researcher in drawing more general conclusions [35,36]. Azungah [37] states that observable arguments are best presented inductively by using people’s perspectives to create broader themes, as well as to generate a theory linking the themes. In this regard, using an inductive approach was appropriate for this qualitative study to allow the researcher to use views from the reviewed sources to build broader themes regarding the aging effect of polymers under UV radiation.

### 2.3. Research Design

The researcher applied an exploratory research design to investigate the aging effect of polymers when exposed to UV radiation. The essence of exploratory research is to establish tentative findings concerning an area of research to form the background for further conclusive research in the future [38,39]. Mbaka and Isiramen [40] and Reiter [41] argue that the use of exploratory research is profoundly suitable in underexplored subject areas to identify explanatory findings addressing the study question. While the concept of the aging of polymers has received extensive research attention over the years, understanding the accelerated effect of UV radiation on their deterioration sparks novel research interest. Such tentative findings were integral in enhancing the understanding of how different polymer composites degrade when exposed to thermal radiation from UV rays. Thus, the exploratory research design was suitable for this study to foster the determination of tentative findings on the aging effect of polymers exposed to UV radiation, and therefore to set the foundation for future conclusive findings.

### 2.4. Search Strategy

The search strategy employed in this study involved the use of database searching, the Google Scholar search engine, and the snowballing technique. In relation to database searching, the researcher used five scholarly databases to obtain academic publications relating to the aging of polymers exposed to UV radiation. The databases used included MDPI, ScienceDirect, Scopus, Emerald Insight, and Sage Open. These databases were used because they contain highly credible journal articles that undergo the process of peer review, as mentioned by Gusenbauer and Haddaway [42]. Database searching was employed as it was considered suitable for enabling the researcher to identify organized and useful scholarly information from published literature, therefore contributing to the rigor since the review articles were selected from highly reputable journals. Bramer et al. [43] contend that creating a suitable search strategy assists researchers in identifying the best balance between sensitive and specific sources to be reviewed. Apart from the database search, the researcher used the Google Scholar search engine to retrieve further sources relating to the study subject. Halevi et al. [44] suggest that the Google Scholar search engine is important in identifying indexed journals, thus providing data for scientific evaluation. Lastly, the researcher also used snowballing to broaden the review materials identified by perusing the bibliographies of the initially selected articles to check whether the cited articles were suitable for this study. According to Wohlin [45], the snowballing search technique is implemented by using the reference list of a few studies to help identify other papers that align with the eligibility criteria. Therefore, this technique was used in this study by checking the reference list of articles identified from the databases mentioned above to identify other suitable references consistent with the inclusion criteria.

The keywords used in the search process were “aging, thermal, conditions, polymers, composites, UV radiation, solar radiation, effect, and impact”. These keywords were merged with Boolean operators “OR” and “AND” to form suitable search terms and phrases to identify relevant review articles. Boolean operators are essential in providing quicker access to articles, as they increase the efficiency of the search process by 77% relative to free-text queries [46]. Table 1 below shows the combinations of the various keywords with Boolean operators to create the search terms utilized in identifying suitable review articles for this study.

Moreover, the review sources were assessed and qualified based on eligibility criteria. For instance, only sources published between 1989 and 2024 were included in the study to comprehensively review scholarly perspectives on the aging of polymers and polymer composites across time. Additionally, qualitative, quantitative, and mixed-methods research articles were included. Moreover, the researcher included articles focusing on the aging of polymers under UV exposure. For this reason, articles discussing the aging of polymers under other environmental conditions were rejected. Further, non-English publications were excluded from this study due to the language barrier. Based on these criteria, a total of 137 articles were selected for review.

### 2.5. Data Analysis

Thematic analysis was applied to analyze the findings. Clarke and Braun [47] suggest that thematic analysis is a flexible and adaptable technique for presenting findings qualitatively. In this regard, the researcher used this analytical technique to identify codes and concepts from the review sources, group similar arguments and assertions, contrast opposing views, and synthesize them to make meaning. The process of analysis followed Nowell et al.’s [48] suggestion, which involves six phases of data synthesis, including familiarization, the identification of initial concepts and arguments, developing themes, refining them, defining them, and producing the final manuscript.

### 2.6. Ethical Considerations

The study complied with the ethical guidelines of conducting secondary research. According to Tripathy [49], secondary research relying on readily available online information should ensure acknowledgment of the authorship of the sources, since permission to use the information they contain is already implied. Similarly, Jol and Stommel [50] mentioned that in secondary research ethics, the aspects of consent, access, and permission are crucial since evaluators must remain consistent within the confines of good research conduct principles. As such, researchers are required to check whether their utilization of secondary data aligns with the consent initially obtained from informants. As such, the researcher referenced all the articles whose information was analyzed in this study. Moreover, the researcher ensured that their use of secondary data observed consent, as originally allowed by the participants, by using only free-access publications. Articles requiring a subscription or the contacting of the authors before using their content were excluded from this study to align with ethical guidelines.

### 2.7. Limitations

The research used only free-access articles, implying that studies that required authors’ or publishers’ permission or subscription were omitted. This feature might have potentially limited the study from comprehensively studying the phenomenon of polymers’ aging under UV radiation exposure by accessing a wider pool of scholarly materials. Moreover, using secondary research methodology might have limited this study from benefitting from new perspectives from primary research participants. Synthesizing data from already published materials does not contribute to the discovery of new knowledge but rather determines and validates best practices suggested by other scholars [51]. Nonetheless, secondary research has been credited for being able to help draw new hypotheses, clarify research questions, and avoid overburdening sensitive populations [52]. Thus, despite the limitations of this secondary research, it helped clarify research concerns about the aging of polymers under UV radiation.

## 3. Results

The synthesis of the findings generated five themes. These themes include the theoretical background of aging, the stability of 3D-printed polymers under UV, methods of improving polymer performance, the effect of polymer aging in real-world applications and agricultural plastics, and experimental methods of determining the aging process of polymers exposed to UV radiation. The literature review matrix is attached in the Appendix A.

### 3.1. Theoretical Background of Aging

This theme emerged from the theoretical discussions advanced by several authors of the reviewed papers regarding the aging of polymers, underscoring the causes, nature, and type of aging for polymeric materials. For instance, Zhang and Yang [53] argue that after the first study on the degradation of polymers was conducted in 1935, researchers in the following decades shifted their attention to systematically examining the stabilization and aging mechanisms of polymeric materials. Experimental results from studies have yielded commendable results, providing vital theoretical positions and simulations of polymer aging [53]. Thus, the aging mechanisms of polymeric materials exposed to thermooxidative conditions, chemical mediators, and photooxidative agents underscore the impact of exposing these products to different environmental conditions on their aging process. Precisely, the reaction aging theory, propounded by Liu and Li [54], indicates that polymeric materials usually obey the process of free radical chain reaction when exposed to the joint action of oxygen, temperature, and light. The aging process is usually initiated because of the homolysis of the molecules’ carbon–carbon or carbon–hydrogen bonds, or the reaction of oxygen with polymeric materials to produce free radicals [55]. Additionally, Day et al. [56] suggested that adding impurities to polymers coupled with initiation effects as well as anti-aging agents can substantially impact their aging reaction. Therefore, the reaction theory of polymer aging suggests that the reaction of polymer molecules’ elements with environmental parameters releases free radicals that facilitate the process of aging. These theories are relevant to this topic as they explain how free radicals are generated and how their reaction with oxygen, temperature, and UV radiation causes polymers and polymer composites to age.

Another theory used in explaining the aging process of polymers is diffusion. Propounded by Troev et al. [57], the diffusion theory of aging posits that when permeating molecules diffuse into a solid polymeric material, they interact with it. This diffusion mechanism can employ either a thermal energy perspective or the free volume view [53]. The thermal fluctuation theory suggests that over time, fragments of polymeric materials absorb energy, loosen, and rearrange themselves, which pushes permeating molecules into novel positions [58]. On the other hand, the free volume theory holds that permeating molecules can migrate by changing positions between polymeric materials’ free holes or volumes [53,54]. According to Arya et al. [59], the diffusion theory holds that minute molecules of polymeric materials diffuse as the temperature and concentrations change. Indeed, Arya et al. [59] assert that free volume conceptualization entails the total mass that polymer chains do not occupy; thus, diffusing molecules are able to occupy it. Solvents raise a polymer’s free volumes as well as the degree of diffusion occurring in its free volume chains [60,61]. The theory posits that the extra, unoccupied total mass in polymeric materials fosters mass transport within the polymer structure [59]. Hence, the diffusion theory views the possibility of the positional change of permeating molecules, displacing free volumes and thereby gradually deteriorating polymeric materials.

Theoretically, polymers chemically react when exposed to UV radiation, causing them to degrade. Henry et al. [62] suggest that the aging of polymers reinforced with carbon fibers during the testing and storage phases is attributed to the impact of light. However, other researchers suggest that even without UV radiations, polymeric materials age as goldish particles are formed on their surface after soft abrasion with cleanroom wipes or foils [53,63]. The goldish particles are epoxy resins that emanate from polymeric materials, thus leading to concerns being raised concerning the integrity of polymers reinforced with carbon fibers [62]. Such degradation or aging can potentially influence the contamination of sensitive surfaces of products made using polymers. Indeed, thermal and hypothermal aging can alter epoxies [53,63]. During the process of aging, epoxy resins undergo diverse chemical reactions, like oxidation, which lead to the production of free radicals [63]. These elements react with oxygen from the atmosphere to form peroxides, which facilitate further deterioration [53]. Essentially, this outcome impacts polymeric materials’ mechanical, optical as well as thermal characteristics. Photooxidation is one such element that can foster the alteration of epoxies when exposed to UV radiations as the energetic wavelengths activate the degradation process.

### 3.2. The Stability of 3D Printed Polymers under UV

The effect of UV radiation on the aging of 3D polymers has been examined by various researchers. For instance, Amza et al. [64] examined the deterioration of polymeric materials exposed to UV radiation to determine those that can withstand band wavelengths of 200 nm to 280 nm as a mechanism for sterilizing parts of personal protective equipment with 3D-printed parts. Amza et al. [64] argued that identifying the polymers that withstand UV radiation of given wavelengths is important in fostering their reusability. However, Amza et al. [64] noted that most polymers degrade when exposed to UV radiation, making it integral to determine 3D materials’ ability to withstand sterilization. For instance, using 3D-printed parts of a polymer, Amza et al. [64] investigated the impacts of accelerated aging by exposing them to UV radiation to determine their mechanical properties. The findings show that the 3D-printed parts of a polycarbonate and acrylonitrile butadiene styrene polymer (ABS-PC) had minimal degradations when exposed to an irradiation procedure [64,65,66,67]. Precisely, the ABS-PC polymer’s 3D-printed parts retained their tensile strength after exposure to the irradiation procedure [54]. However, the irradiated parts of the 3D polymer showed a slight loss of stiffness (5.2%) coupled with a 6.5% loss of compressive strength [64]. Thus, 3D polymers are able to withstand irradiation procedures.

### 3.3. Methods of Improving Polymer Performance

The performance of polymeric materials can be enhanced through additives. According to Franco et al. [68], a polymer’s stability against UV radiation effects is enhanced by adding different UV stabilizers when the materials are manufactured. One such stabilizer is carbon black, which is a particulate added to the surface of polymeric materials to give it mechanical protection by absorbing radiations from UV radiation [69]. However, it is crucial to state that the efficiency of absorption relies on the particle size of the carbon black [70]. In this regard, smaller particles have a larger surface for contact, offering enhanced protection of polymeric materials against UV radiation up to a limit of 20 nm in size. Below this threshold, no extra protective gain can be leveraged [71]. For instance, perspectives from Franco et al. [68] suggest that geotextile protection requires carbon black that has a diameter ranging between 22 and 25 nm. Hence, carbon black and other stabilizers provide polymeric materials with protection against UV radiation, enhancing their performance against thermal conditions.

**Figure 2:**
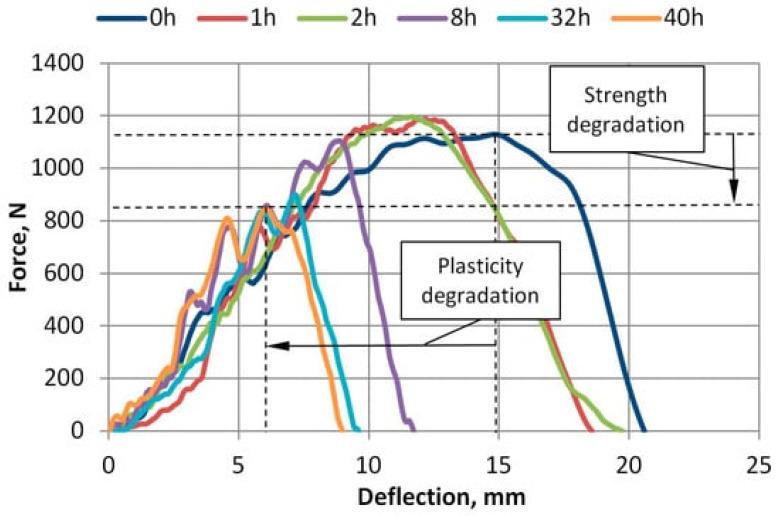
A graph of force vs. deflection following UV exposure of R_A-PET films [75].

Improving the communication structures of polymers is also integral in enhancing their anti-aging performance. A study by Hachicha and Overmeyer [72] explored the possibility of integrated waveguides offering enhanced communication on polymer structures. The authors suggested that such integrated waveguides are embedded on the surfaces to provide optics and relay information relating to the humidity, vibration, and electromagnetic fields of polymeric materials [72]. Using integrated micro-polymer optical fibers (μ-POF), Hachicha and Overmeyer [72] explained that bonding to the surface can help dispense adhesives that dispel UV radiations and the optical waveguide attached to the polymeric material’s end-facets of sender or receiver. A μ-POF is defined as a transmission fiber and fiber optic component used in telecommunication networks [72]. The findings from the study demonstrate that when deployed, the system’s smart parts deteriorate due to mechanical as well as thermal stress [72]. This aging element hampers bonded μ-POFs’ positional stability as well as the efficiency of optical communication. Furthermore, Hoghoghifard et al. [73] have suggested the use of doping and redoping procedures to enhance the effectiveness as well as dielectric features of polyaniline-coated polyester fabric. This method potentially enhances the stability of polymers, hindering them from degradation. According to the authors, employing doping and redoping effectively can help improve polymeric materials’ shielding effectiveness by transforming their surface resistivity as well as dielectric permittivity [73]. Some of the specific material combinations that can be used in the doping mechanism include PANI fabric samples that are flexible, thin and lightweight, as well as with low-resistive features [73]. The PANI fabrics recommended for this material combination to achieve polymer safeguarding through doping are those with an X band frequency range of 8.2–12.4 GHz [73]. As such, fabrics that have coatings of absorber materials like conductive polymers yield flexibility, light weight and efficiency, as well as an antistatic electromagnetic interference (EMI) shield [73]. A similar study by Egghe et al. [74] comparatively investigated plasma-based polymers’ aging behavior vis-à-vis silicone elastomer thin films by measuring their water contact angles after plasma treatments upon reaching their super-hydrophilic states. The findings from this experimental study established that both materials attained similar super-hydrophilic states, water contact angles, and wettability [74]. However, the findings from X-ray photoelectron spectroscopy measurements after eleven days show that manifold aging processes occurred in the silicone elastomer, while the plasma polymerized hexamethyldisiloxane exhibited coating due to the mobility of oxidized short-chain fragments [74]. Thus, 3-aminopropyltriethoxysilane can be applied to the aged coatings to silanize them even though they have distinct stabilities. In contrast, a study by Vasylius et al. [75] examined the mechanical properties of amorphous polyethylene terephthalate (A-PET) films exposed to UV radiation to understand the degradation of these polymeric materials after exposure to UV aging. A-PET is defined as a thermoplastic film manufactured via extrusion from a polymeric material called polyethylene terephthalate [75]. The experimental study established that exposing A-PET films to a UV radiation of 2.45 W/m^2^ for a varied range of durations results in their loss of tensile strength, specifically losing plasticity even after being exposed to solar irradiance briefly [75]. Thus, exposing this type of polymeric material to UV irradiation deteriorates its mechanical properties due to elastic and plastic deformations, particularly for recycled materials [75]. Figure 2 below illustrates the aging graph plots of R_A-PET films after UV exposure.

Based on Figure 2 above, the force and deflection dependence curves of R_A-PET films accelerated at different durations of UV aging demonstrate that the maximum force needed to penetrate the polymer declines after extended exposure. This finding shows that the films substantially degrade when exposed to UV because they lose their tensile strength. In that regard, different polymeric materials have discernibly distinct levels of performance when exposed to UV radiation, which weakens their physical and mechanical properties [76,77]. Nonetheless, diverse approaches can help enhance the performance of polymeric materials against UV irradiation.

### 3.4. The Effect of Polymer Aging in Real-World Applications and Agricultural Plastics

**Figure 3:**
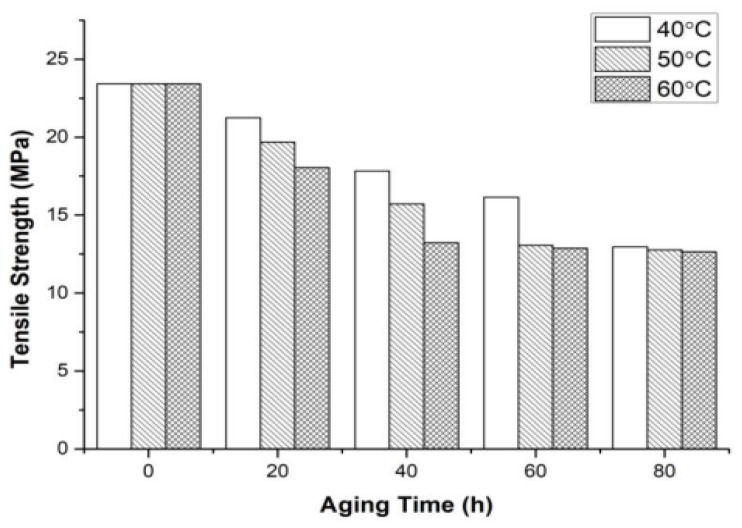
Change in tensile strength of flour/PLA composite after UV aging exposure (source: Lin et al. [81]).

Polymers are used in different industries ranging from construction to everyday applications, such as through the use of plastics for packaging substances and agricultural applications in erecting greenhouse structures. For instance, 3D polymers have also been increasingly used in real-world applications in contemporary industries [78,79,80,81,82,83]. Figure 3 below shows the change in tensile conditions of a flour/PLA composite after consistent exposure to UV radiation.

Based on Figure 3 above, the tensile strength of the initial flour/PLA polymer composite declines after exposure to UV radiation. The legend for temperature ranges shows that group 3 filaments had the highest thermal heating conditions, with 60 °C, and they stabilized at 40 h (Figure 3). At thermal conditions of 50 and 40 °C, the tensile strength of the polymer composite stabilized after being treated for 60 and 80 h, respectively. In that regard, a rise in the aging temperatures led to an increase in the aging process of the polymer. As an illustration, polymer concretes, whose binders are made of polymeric materials instead of cement, are one such area where 3D polymers are used in real life [84]. House et al. [85] note that although 3D printing is linked to the development of asthmatic conditions, it is one of the novel technologies that generate three-dimensional images from digital files by depositing and integrating plastic materials with others like metals and ceramics through additive manufacturing. Polymer concretes have been used continuously across different areas of applications, including making precast architectural facades, wastewater pipes, manholes, and bridge deck overlays, among others [84]. In comparison to traditional Portland cement concrete, polymer concretes have elevated tensile strengths, better bond strengths, and enhanced durability [85]. Bedi et al. [86] note that although more costly than conventional concrete, polymer concrete is reputed to possess an enhanced microstructure, which offers it better durability. Krčma et al. [87] and Furet et al. [88] explain that to create 3D polymer concrete, aggregates, polymer resin, and fillers are mixed with a substituted cement binder. The resulting polymer concrete can thus be used in 3D printing, wherein the stability of the shape is a vital feature. Kozicki et al. [89] mention that 3D dosimeters contain compounds that undergo changes under the effects of ionizing radiation. Chapiro [90], Lebedev and Startsev [91], and Davenas et al. [92] suggest that ionizing radiation influences material aging because its features bring about this change, which is evident in polymers as well as polymer composite materials. In this regard, polymer composite materials have thermoplastic coupled with thermoset matrices that possess glass, carbon, and carbon reinforcements, among other fibers that degrade once exposed to ionization radiation sources [85,93]. However, contrastive evidence from Hakamivala et al. [94] suggests that the integration of individual as well as interactive parameters of 3D printing, like layer thickness, delay duration, and printing orientation, can enhance the scaffolding mechanical features as well as the dimensional error. Integrating these multiple printing parameters into the 3D printing process enhances polymeric materials’ resistance to degradation and aging. For instance, the results from the study’s Response Surface Methodology reiterate that increasing the 3D printing process delay time leads to enhanced binder spreading coupled with uniformity [94]. This outcome is accompanied by the better compression strength of polymeric materials. Moreover, increasing delay time ensures that the binder spreads more vertically, thus generating increased dimensional errors in the Z-direction [94]. Thus, applying the Response Surface Methodology offers a timely and cost-effective design for printing prototypes with enhanced polymer strength as well as dimensional errors.

However, polymer aging impacts the performance and stability of polymeric materials in their real-life applications. In a study by Sun et al. [95], the oxidation and degradation of high-viscosity modified asphalt were examined through gel permeation chromatography to determine the aging and stability features. According to Sun et al. [95] and Lin et al. [96], asphalt plays an important role in constructing pavements; however, the longevity of infrastructures made from this polymeric material degrades faster due to aging. Findings from Sun et al. [95] indicate that extending the proportion of large molecules and decreasing the proportion of polymer weight helps characterize polymeric materials’ oxidation and degradation levels, respectively. Indeed, Makki et al. [97] underscored that the oxidation and polymer degradation degrees for high-viscosity modified asphalt rise as it ages and weathers at its apex. On the other hand, Lin et al. [96] suggested that combining modified asphalt mixtures with fibers can enhance the porosity of pavement construction owing to their superior stability and anti-aging properties. In that regard, Lin et al. [96] reported that polyester fibers combined with asphalt mixtures can show enhanced performance against raveling and fatigue, as well as resisting the rutting and cracking of pavements constructed using these materials. However, such resistance and enhanced performance features were limited when lignin fibers were added to the asphalt mixtures. Both polyester and lignin exhibit improved stability against short-term and long-term aging. The results from the Fourier Transform Infrared Spectroscopy analysis show that adding fiber to bitumen does not yield substantial impacts on its oxidation or deterioration [96]. High-Content SBS Polymer-Modified Bitumen has exceptional features enabling it to develop a masking effect in its original state, thus concealing the impacts of fiber enhancement [95,96,97]. After extensive aging, this impact becomes profound. Thus, Lin et al. [96] recommend that the assessment and design of the performance of high-content SBS polymer-modified bitumen should be determined by post-aging functioning. Likewise, Desidery and Lanotte [98] examined the effects of modifying polymeric materials with asphalt binders on their chemical, thermal as well as microstructural elements. The experimental findings of the study reveal that undisclosed crystalline modifiers had significant effects on the microstructure of asphalt binders [98]. This impact was not established when styrene–butadiene–styrene (SBS) thermoplastic elastomers were used to modify the microstructure [98]. Therefore, polymeric materials’ aging processes impact the features of the base bitumen, undisclosed crystalline modifiers, and SBS. Hu et al. [99] contend that new materials like light-absorbing materials, antioxidants, and light-shielding materials can be uniquely combined and optimized to yield enhanced anti-weather aging for asphalt polymers. Combining these three materials can help improve the high-content polymer-modified asphalt’s anti-aging properties by absorbing UV radiation, shielding it, and neutralizing free radicals [99]. In support, Goncalves Bardi et al. [100] assert that polymerization reactions are used in curing blend substrates, which entails converting reactive formulations into highly cross-linked films, resulting in the creation of a 3D network capable of resisting external degradation factors because physical–chemical reactions cannot undo it. Consequently, this anti-aging property is crucial in preventing polar oxygen-containing functional groups from forming, as well as preventing the deterioration of polymer molecules when exposed to extreme weather conditions.

**Figure 4:**
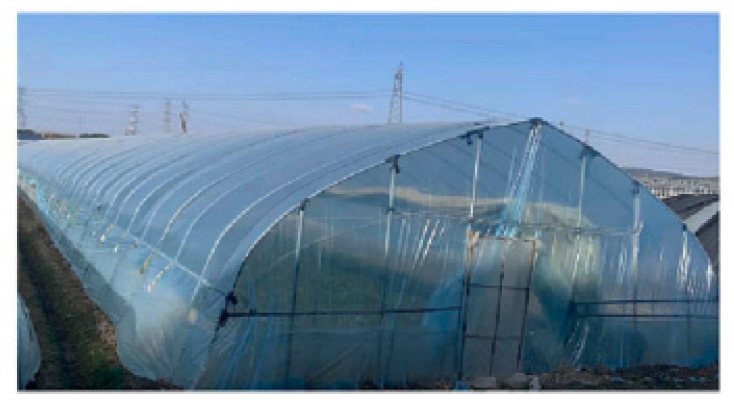
Illustration of greenhouse use of plastics [101].

Polymeric materials are also used in agriculture to promote crop productivity. In greenhouses, plastics are used to increase crop yield by shielding plants from adverse weather conditions. Figure 4 below shows a greenhouse using plastics to regulate the amount of light penetrating for crop production.

UV radiations of the size 200–400 nm can harm plant growth [101]. In contrast, plant growth is promoted by blue-violet and red-orange light, which is made possible through the conversion of UV radiations in greenhouses. In concurrence, Vijayalakshmi et al. [102] argue that plastic greenhouses are a typical application of polymers in agriculture as they help convert harsh UV radiations to bluish-violet light of between 400 and 480 nm and reddish-orange light of between 600 and 700 nm. These plastic materials are able to absorb a portion of the light spectrum generated by the Sun and convert it into the needed light spectrum to increase agricultural productivity. Higher UV radiation wavelengths are scarcely absorbed by chlorophyll. As such, plastic greenhouses show extensive application in cold climates to convert UV photons to bluish-violet and reddish-orange light, which is vital in using electromagnetic energy in cultivating plants [102]. Research into this area by Wang et al. [101] suggests that single-light agricultural films can be used to regulate infrared light in greenhouses. In their study, Wang et al. [101] created a composite coating of waterborne polyurethane (WPU) to convert light and bar light for greenhouse films, thus underscoring how polymers are used in greenhouse production to promote a rise in crop yield. In this regard, polymeric materials can be utilized as radiation conversion materials to convert UV radiations to favorable light that promotes plant growth. In contrast, Al-Helal et al. [103] suggested that under intense climatic conditions, plastic-covered greenhouses lose their optimal properties very quickly due to aging, leading to a damaged orientation and shape. It is worth emphasizing that the degradation rate of greenhouse plastics depends on the surface location as well as the underlying orientation of the cover [103,104]. Thus, the degradation of greenhouse plastics impacts the longevity of use in arid climate conditions due to high temperatures that denature their amorphous features.

Different soil and humidity conditions influence the accelerated aging of agricultural microplastics differently. A study by Bonyadinejad et al. [105] examined the phenomenon of the photodegradation of microplastics used in agricultural production after their accelerated use to determine aging when exposed to environmental conditions. The photodegradation behavior of low-density polyethylene (LDPE) microplastics was studied via accelerated UVA radiation experimentations under varying conditions of relative humidity as well as soil deposition [105]. Two humidity conditions of RH10 and RH70 were used in that experiment, while the degree of UV radiation with long wavelengths (UVA) was calculated through the spectral quantum yield [105]. The findings underscored that LDPE products with a lower molecular weight of Mw = 233 kD underwent greater photodegradation than heavier ones with Mw = 515 kD [105]. High humidity constrains microplastics’ photooxidation process and reduces surface changes in these polymeric materials [106,107,108,109,110,111,112,113,114,115]. However, soil particles’ deposition impacts microplastics’ photodegradation behavior [105,114]. As such, microplastics covered by soil particles do not experience degradation, as opposed to those deposited near soil particles [105,112]. Hence, covering microplastics with soil particles is integral in extending their lifespans for agricultural use. Indeed, an experiment conducted by Amin et al. [109] on the effect of poly-starch N on the natural weathering of LDPE indicated that when these two components are blended, the matrix reduces the tensile properties and extent of crystallinity of the polymer. Increasing the proportion of poly-starch N in the blend heightens the decline of the polymer’s tensile properties [109]. However, Amin et al. [109] demonstrated that exposing the blends to natural weathering processes and increasing the volume of Polystarch N in the mix exacerbates natural degradation, as revealed by the scanning electron microscope tests that were conducted. Thus, blending LDPE with Polystarch N increases polymer degradation and aging.

Furthermore, agrochemicals also accelerate the aging process of plastics used in agriculture. For instance, findings from Picuno et al. [116] and Schettini et al. [117] have also demonstrated that when exposed to varying agrochemicals like anti-aphid or fungicides and aged artificially for distinct periods, agricultural plastic film is contaminated and its lifespan reduced. In this regard, agrochemicals considerably impact plastic films by worsening their aging processes and rapidly reducing their mechanical properties, thereby reducing their lifespans by above 50% relative to virgin plastic [116,117]. This aging phenomenon of polymers used in agriculture has twofold impacts, lowering the plastic films’ working age and reducing their potential transformation into closed-loop recycled materials after entry into the recycling stage [112,118]. Indeed, when these plastic films interact with agrochemicals, they tend to degrade faster, as confirmed by the rise in the number of detected carbonyl indexes (CI) [116]. This aspect suggests the impossibility of recycling plastic film that has come into contact with agrochemicals during its useful working life [116]. Nevertheless, high thermal conditions, humidity, and the addition of impurities such as agrochemicals accelerate the aging of LDPE polymers used in greenhouse applications [119]. For instance, Dehb et al. [120] and Dehb et al. [121] underscored that the degradation of carboxyl groups in many polymers requires extensive exposure to a UV irradiation source for their aging to be observed during field studies and laboratory tests. LDPE degradation was one of the polymers reviewed in this study concerning its degradation and aging process, and the findings suggested that it deteriorates once exposed to a UV radiation source for extensive hours [122]. Electromagnetic sources with wavelengths ranging between 300 and 400 nm could influence the generation and breakage of the bonds between polymer molecules [123,124]. Free radicals are produced in the polymer as the wavelength of electromagnetic radiation increases towards the 400 nm threshold [125]. The adverse effects of plastic material aging on the generation of secondary materials like anthocyanins and total phenolics that degrade the surroundings have also been covered by Katsoulas et al. [126] as one of the elements of pure polythene used for blocking UV radiation in greenhouses. Thus, the aging effect of polymers is experienced by farmers employing greenhouses to increase crop yield, as the adverse thermooxidative conditions contribute to the aging of plastic materials employed.

Polymers are also used in other large-scale industrial uses. For large-scale industrial applications, Al-Salem et al. [127], Feldman [128], and Andrady et al. [129] argue that fillers and reinforced polyolefin (PO) polymers have diverse applications, including in traditional wood fibers, construction, polyesters, and short glass production, and their contemporary uses continue to rise. Some of the contemporary applications include engineering disciplines and customized use areas like protection surfaces and insulators [103,119,130]. However, accelerated weathering tests conducted by Al-Salem et al. [131] on linear low-density polyethylene (LLDPE) blended with plastic films confirmed that extreme temperatures degrade them faster, as polyolefin polymers lose their amorphous region once exposed to UV radiation. UV radiation deteriorates plastic waste components, accelerating their rate of aging [132,133,134]. Likewise, previous research by Ávila-López et al. [135] and Cacuro et al. [136] acknowledged that many polymeric materials are vulnerable to UV radiation because the overall energy of UV radiation supersedes the strength of the inherent carbon bonds in polymers. Concurrent perspectives from Garg et al. [137], Lei et al. [138], and Palkar et al. [139] indicate that electromagnetic radiation from a UV source can fracture a polymer and reduce its molecular weight, leading to the generation of free radicals. Moreover, Cheng et al. [140] and Fraga Dominguez et al. [141] suggested that polymer irradiation from a UV source can lead to an increase in the molecular chain length coupled with its cross-linking. However, Rivas Aiello et al. [142] and Tian et al. [143] underscored that different polymeric materials may need exposure to different aging conditions to facilitate the degradation and deterioration of their mechanical properties. In contrast, studies by Xiu et al. [144] and Zhou et al. [145] have illustrated that titanium dioxide (TiO_2_) can be added to Polylactide (PLA) to enhance its UV resistance for outdoor applications. In concurrence, Smith et al. [146] have suggested the use of Porosity Induced Side chain Adsorption (PISA) as a pathway for enhancing super-glassy polymers’ stability against physical aging by using the porous aromatic framework PAF-1 to improve the void space, enhance gas transport speed and freeze glass polymers in a state of low density. Likewise, El-Hiti et al. [147] suggest the addition of UV absorbers such as polyphosphates, organometallic complexes and Schiff bases as plastic photostabilizers to provide a mechanism for modifying polymeric materials’ resistance to aging. Furthermore, to circumvent the photodegradation of polymeric materials’ mechanistic complexities, Zemke et al. [148], Auras et al. [149], Wallnöfer-Ogris et al. [150], Karlsson and Albertsson [151], He et al. [152], Ray and Cooney [153] and La Mantia et al. [154] have suggested the use polymers that possess metal–metal bonds integrated into their backbone. Irradiating these materials breaks the metal–metal bonds, followed by their radicals being trapped by a suitable radical trap like molecular oxygen or the bond between carbon and chlorine molecules [148]. This phenomenon leads to the creation of a net backbone cleavage, which deters photodegradation [148]. In this regard, there are different methods through which plastics used in agricultural applications can be made to last longer.

### 3.5. Experimental Methods of Determining the Aging Process of Polymers Exposed to UV Radiation

This theme emerged from views and findings from extant literature about the various techniques applied to gauge the aging process of polymeric materials under UV radiation. The aging of polymeric materials has been measured through different techniques, depending on the type of polymer and the conditions to which it is exposed. For instance, Ricci et al. [155] and Hebert and Ediger [156] assert that optical probe reorientation coupled with mechanical stress relaxation is used to measure aging in PLA and glassy poly(methyl methacrylate) (PMMA) through their segmental dynamics. PMMA is defined as a synthetic polymer utilized as a lighter and shatter-resistant alternative to glass in diverse applications like windows and aquariums [155]. These experiments usually last for 8 h and are conducted in a linear response regime, with a heat range of 6 to 30 k underlying the glass transition temperature (Tg) [155]. In all these experiments, correlations are conducted over the observation timelines and connected by a power law having an exponent of about 1. Moreover, concerning thermal techniques for polymer aging, Hodgson et al. [157] suggested that polymer analysis, polymer characterization, and the impacts of thermal treatments on their molecular orientations are acknowledged in the extant literature. For conjugated organic polymers, Han et al. [158] suggest the establishment of D-A type conjugated polymers to foster electron transfer via the polarization impact from the donor to the acceptor. This method promotes excellent electron migrations that are integral in enhancing photoconductivity as well as photocatalytic activity.

**Figure 5:**
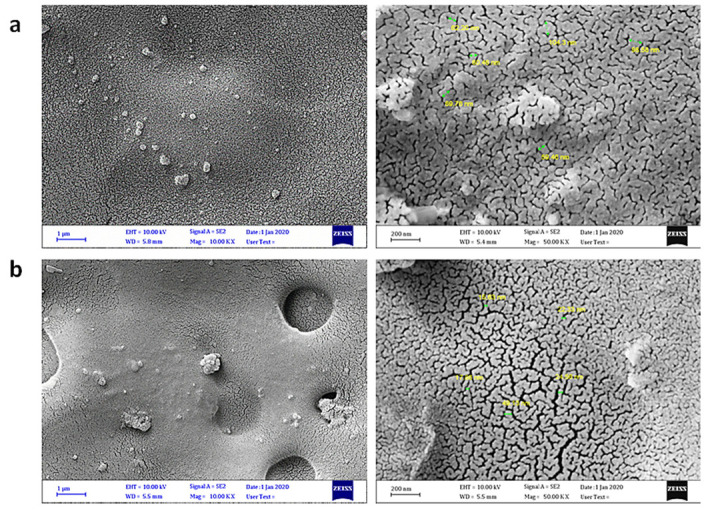
Results of field-emission scanning electron microscopy of different scales between (**a**) and (**b**) [160].

Additionally, experimental studies examining photodegradation have used nano-TiO_2_ and surfaces modified with polyaniline (PANI) to determine the aging properties of polymers and polymer composites. PANI is a polymer composite and blend with diverse applications in fields such as the manufacture of sensors and biomaterials [159]. For instance, Jose et al. [159] examined polystyrene’s (PS) degradation mechanisms using no TiO_2_ and PANI and developed an X-ray diffractogram to disclose the influence of the robust molecular forces between TiO_2_ and PANI on the declined optical bang gap energy between the TiO_2_ and PANI polymer composites. Experimental studies focusing on the effective UV absorbers used to elongate the lifespan of polymers have also been conducted. For instance, an experimental study by Mohammed et al. [160] suggested the use of Valsartan Tin Complexes to shield the surface of Poly(Vinyl Chloride) films from extreme irradiation and subsequent degradation. Figure 5 below shows the experimental results of the latter study’s irradiation.

**Figure 6:**
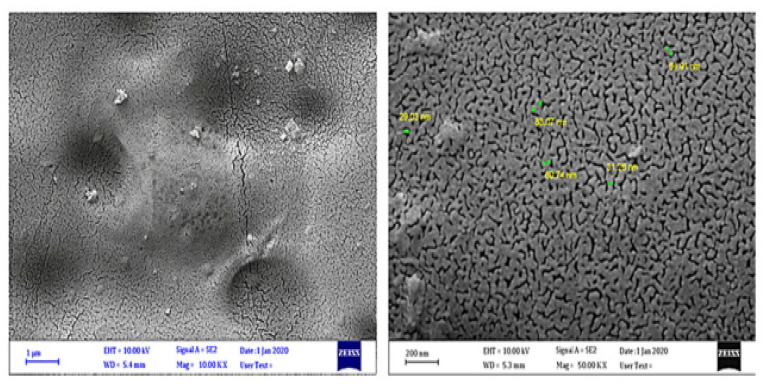
PVC after UV irradiation plus four complexes [160].

Figure 5a above shows the image of the plastic before irradiation, while (b) shows the polymer after irradiation. This experimental study used Fourier-transform infrared spectroscopy, the determination of molecular weight, and the computation of weight loss to identify the protection properties of valsartan complexes on the photoirradiation of films. Figure 6 below shows the image of the polymer after UV irradiation.

**Figure 7:**
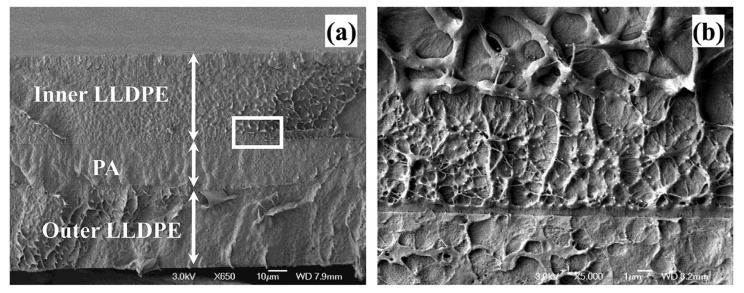
Image of the aged LDPE film in (**a**), and higher scale the framed part in (**b**) [106].

Irradiating the PVC film for extended durations led to the breakage of polymer bonds and the formation of rough as well as broken surfaces (Figure 4). Adding four complexes to the polymer and exposing the polymer to 300 h of UV radiation enhanced the photostability of the films [160]. For the degradation of the SPS polymer, Ding et al.’s [161] experimental research used confocal laser scanning microscopy (CLSM) to characterize morphological changes after exposure to UV radiation. The study considered various protocols of aging, including accelerated laboratory degradation and aged samples gathered from the field with varying in-service periods [161]. The study then processed the polymer’s scanned images in the 2D phase at various depths, followed by 3D reconstructions, which helped derive polymer morphology indices. The findings from the study disclosed that as aging progresses, polymer particles transform from considerably large ellipsoidal shapes to smaller spherical shapes [161]. Increasing aging temperatures resulted in accelerated polymer acceleration at given rheological levels [161]. Moreover, melt viscoelastic assessment studies have also been conducted to determine the aging properties of poly(lactic acid) by burying polymer films in composts at 58 °C to determine their biodegradability and assess their molecular evolution [162]. Indeed, exposing polymer composites to UV radiations of band wavelengths of 290–400 nm makes their epoxy matrices age rapidly and extensively, thus deteriorating their mechanical properties [163,164,165]. These experiments are conducted through the use of UV lamps in the laboratory. The transformations in the physical as well as chemical properties during polymer aging are often monitored through different experimental methods like absorption spectroscopy, which uses infrared and ultraviolet–visible changes to gauge the degradation of polymer characteristics [166]. Other methods include measuring alterations in average molecular weights of polymers and their polydispersity using Gel Permeation Chromatography, conducting microscopic analysis through optical and scanning electron microscopy, and performing tensile measurements [167,168,169,170]. Furthermore, standard laboratory tests can be conducted to determine the biodegradation of plastic material [171,172]. Nonetheless, [173] underscored that UV radiation severely deteriorates the network structure established by the cross-linking impact in SBC-modified asphalt binders. Findings from Kyrikou et al. [174] and Briassoulis et al. [175] suggest that UV radiation can fracture a polymeric material, leading to a decline in its molecular weight as well as the generation of free radicals. Maraveas et al. [176] suggested that for film-based polymers, UV aging impacts their tensile strength significantly, thus causing a faster degradation of their underlying mechanical properties. Results from Maraveas et al. [177] suggest that exposing polymeric materials to UV radiations influences their photooxidative aging, resulting in the deterioration of polymer chains. This aspect, therefore, produces free radicals and influences a reduction in the polymer’s molecular weight [178]. Figure 7 below shows an image of an aged agricultural polymer.

**Figure 8:**
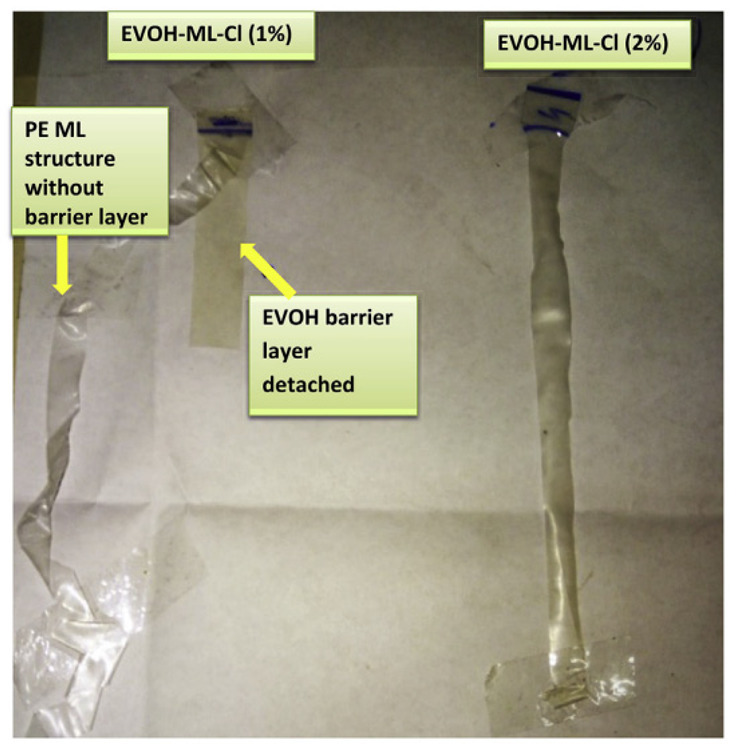
Combined impact of UV radiation and agrichemicals on polymer aging [175].

Figure 7a illustrates a cross-section of the aged polymer that has deteriorated after extensive exposure to UV radiation. Part (b) captures an image of the framed area, indicating the impact of extended UV radiation on agricultural polymer degradation. When UV radiation and agrochemicals are combined, they have an effect on polymer aging that is demonstrated in Figure 8 below using ethylene vinyl alcohol (EVOH):

As illustrated, the left diagram indicates the degradation of EVOH after exposure to intense temperatures of 100 MJ/m^2^, while on the right, the film’s structure after the impact is depicted in Figure 6.

Essentially, the reduced molecular weight underpins polymers’ aging process once exposed to UV radiation [179,180]. Moreover, the thermal degradation of PLA via pyrolysis is a complex process that entails the scission of a random main chain and the unzipping of the depolymerization reaction [181,182]. Moreover, the review conducted in this study indicates that the aging of plastics is susceptible to physical, chemical, and biological impacts, which weaken the mechanical properties of polymers [183,184]. These factors constitute natural conditions that are prevalent in most ecosystems, implying that they can comprehensively influence the aging of polymeric materials in their immediate environment when the number of oxidative and thermal elements increases [185,186]. Table 2 below shows some of the widely used polymers and their market shares.

Therefore, as Table 2 above shows, some polymers have a large market share, implying their broad economic use, like PE, while others have a less significant market share, like PS. Thus, determining the aging properties of these polymers is crucial to understanding their expected useful life after constant exposure to UV radiation. Exposing the epoxy resins of polymers to extreme thermal conditions beyond the glass transition temperature and in oxidative environments leads to their thermooxidative degradation [187]. This aspect entails the fragmentation of the crosslinked epoxy’s chemical structure in part due to the carbon-based polymer structure reacting with oxygen molecules [188]. This thermooxidative aging has a central effect in that the polymer initially loses weight during exposure as a consequence of losing moisture and residual volatiles [189]. After exposing the polymer to oxidative and thermal conditions for many hours, it stabilizes its weight, and any further weight loss indicates the generation of gaseous by-products [186,189]. Nonetheless, experimental methods of aging have more pronounced impacts on polymer deterioration during the early phases relative to field aging processes.

## 4. Discussion

The interpretation and discussion of the findings established in the previous section are presented here to demonstrate how the current research objectives were addressed in the review conducted and to contextualize the results within the broader body of knowledge.

### 4.1. Interpretation and Implications of the Findings

The evaluation of the results obtained from the review of the selected sources is presented in terms of the three study objectives.

### 4.2. The Key Theoretical Positions on the Range of the UV Radiation That Affects Polymers

The phenomenon of polymer aging under electromagnetic radiation was explained in this study from the perspective of two theories. Generally, the reaction agency theory is based on the notion that polymeric materials normally follow the process of free radical reactions when they are exposed to a thermooxidative environment. This theory suggests that polymer aging results from the homolysis of the carbon–carbon or carbon–hydrogen molecules, leading to the generation of free radicals. This theoretical explanation mirrors the perspectives advanced by Yousif and Haddad [3] and Kockott [7], indicating that free radicals are responsible for polymer aging as they react with environmental conditions. The other theoretical perspective advanced by scholars posits that permeating molecules interact with the free volumes in solid polymeric materials once they diffuse into them. This migration aspect makes polymers exhibit changes in their mechanical properties, leading to degradation.

The findings of this study have indicated that polymer aging is determined by its stability, and the degree to which such materials have integrated other additives to give them stability and shield against the adverse impact of UV radiation. This result implies that in order to elongate the lifespan of polymeric materials, practitioners must identify suitable additives and use them to stabilize their ability to withstand environmental conditions. The results indicate that polymers degrade faster when exposed to a combined influence of oxygen, high temperatures, and light. This result aligns with the findings established by White [2] and Yousif and Haddad [3], which indicate that exposing polymeric substances to thermal conditions weakens their mechanical properties and degrades their chemical properties over time. Once polymers are exposed to external environmental conditions, they gradually lose their toughness, tensile strength, sensitivity, and reactivity towards chemical substances that are aggressive. Consistent with this result, Qi et al. [22] reported that polymer composites are easily oxidized, softened, and hydrolyzed when exposed to extreme thermal conditions, thus manifesting reduced stiffness and strength. This result implies that for polymers to have extended lifespans, they must be shielded from the adverse impacts of environmental conditions that induce aging.

**Figure 9:**
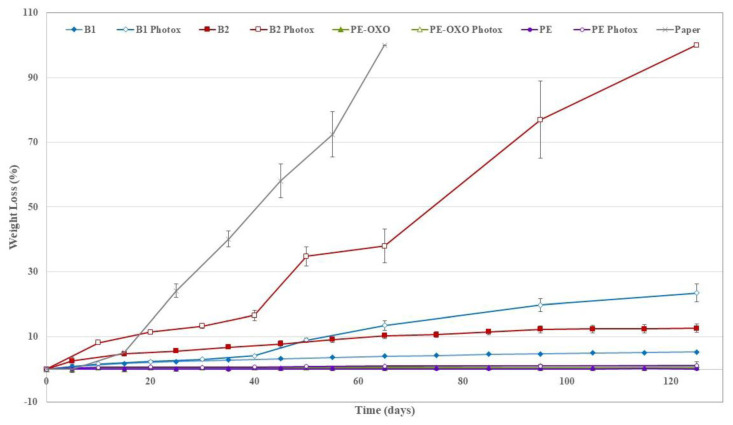
Aging of polymers under UV radiation (source: La Mantia et al. [184]).

The results indicate that different polymeric materials espouse discernible reaction properties when exposed to UV irradiation that help us ascertain their aging characteristics. The graph shown in Figure 9 below indicates this aging acceleration.

As shown in Figure 9 above, the irradiated polymer samples showed an accelerated loss of weight as the number of days increased. This phenomenon implies that UV irradiation accelerated the breaking down of polymers into monomers and oligomers through the random scission of the main chain, which increased as the rate of weight loss increased [184]. For instance, the reviewed studies suggest that exposing film to UV radiation makes it rapidly lose much of its plasticity. As the use of plastic materials for various functional roles spans various industries, it is integral to enhance their stability to yield more economic value by having them degrade at the end of their useful lives [20,21]. Even a slight exposure makes film susceptible to degradation as it significantly loses its mechanical properties following an interaction with thermal energy. This perspective implies that practitioners using film polymers in thermal molding face substantial challenges in ensuring they are active for prolonged durations. In concurrence with this result, Kockott [7] explained that due to inherent economic constraints, testing procedures have been limited to reduce the durations for testing. Nonetheless, products like those used for packaging purposes might crack or tear during the process of manufacturing because the film used loses plasticity once it interacts with UV radiation [7,8]. Indeed, the results underscore that exposing A-PET film to a UV radiation source for between 1 and 2 h and a thermal intensity of less than 2.45 W/m^2^ significantly influenced the deterioration of its plastic properties, emphasizing the need to protect films applied in the thermoforming of packaging materials from direct UV sources. Therefore, irradiating plastic materials for a few hours by exposure to a UV radiation source can potentially degrade their mechanical properties.

Furthermore, the study indicated that the epoxy resins broadly applied in adhesives, coatings, paints, electrical devices, and medical implants are thermosetting polymers exhibiting a similar action to other polymers once exposed to UV radiation. Based on post-exposure results, observable changes are used to inform practitioners of the physical as well as chemical changes that have occurred in a polymer’s mechanical properties. For instance, color change is an observable change that occurs in the polymer’s surface texture as well as crack density once it is exposed to a UV radiation source, thus indicating thermooxidative degradation. Consistent with this result, Qi et al. [22] and Zhumadilova et al. [23] reported that exposing polymer composites to hydrothermal environments accelerates their degradation as they are oxidated, softened, and hydrolyzed faster, leading to color and thermal execution changes. Moreover, the results of this investigation show that alterations in the glass transition temperature are frequently noted during thermooxidative degradation, and they result from additional network crosslinking. Polymeric materials’ performance changes are attributed to exposure to thermal-oxidative conditions like heat, moisture, UV radiation, and precipitation [23,24]. Lastly, the study indicates that fracture toughness increases during extended thermooxidative exposures as a result of chain degradation, which successfully plasticizes the resin. Similar findings were reported by Cosnita et al. [20] and Ghosh et al. [21], indicating that with the persistent rise in the outdoor applications of polymers, it is increasingly vital to understand their precise aging phenomena so as to improve how they are used in practical contexts. This observable change distributes the flawed sites evenly within the polymeric material so that a substantially greater volume can absorb energy. These observations are equally noted for thermosets that are exposed to high temperatures with oxygen, although the degree of degradation is smaller for a given duration of exposure. Therefore, aging in polymers is observed in terms of changes in both the physical as well as chemical properties.

### 4.3. The Testing Procedures Used for Polymer Aging Evaluation and Real-World Applications

The results also reveal that the experimental testing procedures for polymer aging are complex and usually conducted through both fieldwork and lab assessments. Consistent with this result, Arhant et al.’s [15] experiments into polymer durability often took about three years, which experiments, although shorter than the actual in-service durations for plastic materials, are complex and procedural. The results reiterate that these experimental procedures involve the exposure of polymer samples in controlled environments, with increased temperatures, to gauge their deterioration rate. This result aligns with the perspective of Kockott [7], which suggested that polymer aging can be determined through chemiluminescence, which entails applying photooxidation or thermal oxidation to release the weak light utilized in promoting polymer aging. Ideally, this reaction terminates two peroxy radicals, influencing the emission of radiation by an excited carbonyl group that has regained its natural state [7]. Thermal conditions exacerbate the rate of polymer degradation as determined by experimental procedures using different methods, such as optical probe reorientation combined with the relaxation of mechanical stress, the determination of D-A, polymer analysis, and characterization. This result concurs with the findings established by Frigione and Rodríguez-Prieto [5] and Tocháˇcek and Vrátníˇcková [4], which revealed that almost all aging procedures for polymeric materials utilize radiation from xenon lamps, metal halides, and fluorescent lighting tubes. These lighting sources are integral in catalyzing the degradation process of polymers in simulated settings. The findings also underscore that tests for polymer aging through photodegradation employ a combination of nano-TiO_2_ and PANI, followed by an X-ray diffractogram. Consistent with this result, Pickett et al. [6] revealed that the degradation mechanisms for polymers require irradiation as the wavelengths and radiative energies break the chemical bonds binding polymer molecules. Principally, photochemical processes foster polymer degradation by weakening their mechanical properties. However, the findings of Baklan et al. [17] indicate that the rates and grades of polymer degradation may vary due to the heterogeneity of their chemical compounds, as some have hydroxyl groups, while others have oxygen-containing elements and carbonyl. Nonetheless, electromagnetic radiation from the Sun’s rays contributes to the aging and degradation of polymers and polymer composites. This process of degradation affects the useful lifespan of polymer materials used in various industries, ranging from the transport sector to agricultural production. As a result of accelerated aging, when exposing polymers to UV radiation, practitioners usually integrate UV-resistant materials into their structures to help shield them from excess radiation effects. Some of the materials used in this regard include additives, those with metal–metal bonds, and stabilizers, which help reduce the adverse impacts of these radiations.

### 4.4. Available Technical Methods for Improving the Performance of Polymers under UV Radiation

Improving polymer performance is integral in enabling these materials to resist UV-induced degradation. Due to the adverse impacts of UV radiation on the performance of polymeric materials, this study identified various methods used in providing a technical shield effect to prolong their lifespans and counter aging for a considerable period. Consistent findings by Celauro et al. [25] indicate that bitumen modified with polymers is exposed to short-term aging due to the extreme processing temperatures for polymer composites. For instance, the findings of this study underscore the potential detriments of polymer aging as regards the longevity of plastics used in greenhouses. Thus, to elongate the useful lifespans of these plastics, practitioners embed stabilizing features that help protect plastics used in such greenhouses from UV radiation, thereby boosting agricultural production. As a result, the study suggested the use of coatings on the inner side of films used in greenhouses to act as barriers to infrared rays. These coatings also help maintain heat in greenhouses, hence regulating the thermal conditions within the areas where the film is used.

In addition, the results underscore that using additives can help improve the technical performance of polymeric materials by shielding them from degradation once exposed to a UV radiation source. This method entails using different UV stabilizers during the process of manufacturing polymeric materials. This perspective aligns with Seguchi et al. [8], who maintained that thermal oxidation is the most effective technique for stabilizing polymers. This aspect employs stabilizers that are active through thermal conditions to help shield polymers from degradation. For instance, the results suggest that carbon black has been predominantly used as a stabilizer, which is added to the surface of polymers to provide them with mechanical protection by absorbing UV radiation. The efficiency of the absorption depends on the size of carbon black used on the surface of the polymers, with smaller particles providing enhanced protection of the polymers against UV radiation. This method of using carbon black as an additive has been particularly widely used in geotextile protection, reiterating the broader role of stabilizers in enhancing the performance of polymers, particularly those with widespread industrial applications. The results also suggest that 3-aminopropyltriethoxysilane can be added to aged coatings to help silanize them, although they have discernible stabilities. However, as Baklan et al. [17] explained, the rate as well as grade of polymer degradation rely on different factors such as chemical composition, the type of additives applied, surface structure, and the presence of UV-sensitive functional group molecules. Nonetheless, using additives in polymer stabilization is crucial in inducing UV-resistant capabilities in the structures of plastic materials.

The results suggest that polymers’ performance against UV radiation can also benefit from enhanced communication structures, which elevate their anti-aging properties. Similar findings by Moraczewski et al. [18] and Kockott [10] recommend the use of anti-aging compounds to safeguard polymers from adverse UV radiations that cause aging, emphasizing the potential use of plant extracts to enhance aging resistance in polycaprolactone, thereby allowing polymers to be resistant to aging [18]. Some of these anti-aging components can be obtained abundantly from the natural extracts of coffee, cocoa, and cinnamon [18]. However, the results suggest that integrating waveguides in polymeric materials improves their communication, providing an enhanced understanding of polymer structures. Such waveguides are incorporated into the surfaces of polymeric materials to offer them optics and the ability to relay data concerning their humidity, vibration, and electromagnetic fields. Similar results derived by Zhu et al. [26] suggest that modifying polymers with anti-aging components helps them enhance their physicochemical properties, and therefore, makes them able to withstand extreme thermooxidative conditions. For instance, the results underscore that μ-POF can be bonded to the surfaces of polymers to enable them to dispense adhesives, which shield them from UV radiations. Optical waveguides are attached to the end facets of a polymeric material, assisting in relaying information to the sender or receiver concerning the underlying conditions. Furthermore, the results underscore that practitioners use doping and redoping procedures to improve the effectiveness as well as dielectric properties of polyaniline-coated polyester fabric. This method is vital in improving polymers’ stability, which is crucial in preventing them from degrading. Thus, these procedures help transform the resistivity of polymer surfaces as well as their dielectric permittivity.

### 4.5. Research Gaps

There are literature gaps concerning the biological remediation of polymer composites exposed to short wavelengths of UV irradiation (UV-C), which is filtered by the atmosphere. Amza et al. [65] note that UV-C testing standards have not been adequately defined, and regulations about how this technology is used are currently limited. Further research can examine these gaps, focusing on developing an enhanced understanding of this relationship. Moreover, the literature has not extensively studied the specific impacts of UV radiation on polymers with subtly different or discernibly distinctive features. Efforts by past researchers such as Yu et al. [186] and Kaczmarek et al. [187] have acknowledged this dearth in the literature. While these scholars have substantially contributed to understanding the phenomenon of asphalt binder aging under UV radiation exposure, there are notable knowledge gaps in this area of inquiry. Noticeably, asphalt binder components greatly differ in the complexity of their aging process, implying a persistent lack of clarity. Indeed, Yu et al. [186] noted that scholarly advancements have not led to the creation of a standardized evaluation system for asphalt binders, leaving significant gaps requiring scholarly inquiries to bridge. Moreover, future research should explore the environmental impact of chemicals used to delay polymer aging. Furthermore, current studies examining the UV aging of polymeric materials primarily focus on base polymers, while few studies have focused on the specific aging behavior of polymer composites [188,189]. In this regard, further research is necessary to assess the macroscopic features and the microstructure of polymer composites, especially in SBS-modified asphalt binders, plastics used in greenhouses, and those with widespread industrial applications.

### 4.6. Future Research Needs

Future research should examine polymer aging by taking into consideration the unique properties of different polymeric materials and suggesting the most effective stabilizers for polymers with large-scale industrial applications. Such a study could determine the likely methods for enhancing the stability and lifespan of polymers used in big industries such as construction. New approaches need to be determined for determining polymer aging by integrating manifold changes in polymer properties like topographic structure, thickness, break elongation, moisture management, and bursting forces. Such a study could help determine the potential impact of polymer thickness on its degradation upon exposure to UV radiation. It is also important to determine the potential effects of polymers on their surroundings after degradation using life-cycle analysis. Lastly, future research should determine the aging properties of polymers under longer exposure to UV radiation. Such suggested future research will help identify the periods for which polymers can withstand UV radiation before their mechanical properties begin to weaken, and compare the aging of specimens exposed to controlled degradation parameters. Another notable area worth future research focus is determining how distinct soil components compete with microplastics in the adsorption of heavy metals and the overall impact of this reactive behavior on polymer aging. Moreover, further research should determine how aged microplastics contribute to environmental degradation and explore possible pathways for overcoming this externality.

## 5. Conclusions

This research examined the aging process of polymers when exposed to electromagnetic radiation. The study examined three study objectives, including reviewing the key theories underpinning the aging of polymers under UV radiation, examining testing procedures used to evaluate polymer aging and the real-world applications of polymeric materials, and assessing the various methods through which the performance of polymeric materials can be technically improved. The study utilized a literature review methodology to determine answers to these underlying study concerns.

The results indicate that the phenomenon of polymer aging is theoretically understood from the perspectives of the diffusion theory and the reaction aging theory. The analysis of published literature underscores that the reaction aging theory is premised on the assumption that polymers usually experience a free radical chain reaction process, suggesting that by exposing polymeric materials to environmental conditions, free radicals are emitted. Indeed, the results underscore that a combination of environmental factors like light, oxygen, and temperature fosters this reaction, leading to free radical generation. On the other hand, the diffusion theory is premised on the notion that molecules that permeate the polymeric material cause aging through either thermal impact or occupying free volumes. Notably, the theory argues that from the point of view of thermal fluctuation, over time, polymeric material fragments absorb energy, become loose, and reorganize themselves. This process pushes permeating molecules to new positions, leading to the degradation of polymer materials. Moreover, from the free volume perspective, the diffusion theory suggests that permeating molecules migrate by altering their positions within free holes. This migration occurs as the temperature and concentrations of molecules change, hence the diffusion phenomena change too. Therefore, theoretically, the aging of polymers emanates from changes in the physical and mechanical properties of polymeric materials. These changes are observable from the physical as well as chemical changes of polymeric materials exposed to UV radiation and other thermooxidative environments, as they differ in size and structure from the initial material.

Additionally, this study established that various procedures are used when testing for polymer aging, depending on the type of material involved and the method employed. The findings indicate that various experimental methods are used to determine the aging properties of polymers. These procedures include both those conducted in labs and fieldwork. Furthermore, the study reveals that the type of polymeric material being tested and conditions of exposure influence the choice of the experimental procedures employed. In this regard, the findings stress that the optical probe reorientation is combined with mechanical stress relaxation when measuring the aging phenomenon in PLA. Segmental dynamics are thus employed for a period of 8 h, and the time taken for the polymer to transform its chemical and physical properties is determined. Heat is usually regulated during such procedures in the range of 6–30 k below the temperatures for glass transition. Moreover, the results highlight that for testing the aging of conjugated organic polymers, scientists determine the D-A related to the transfer of electrons from the polar elements. In addition, for determining the aging properties of polymeric materials through photodegradation, the results of this study indicate that nano-TiO2 and PANI are used. This experiment usually requires an X-ray diffractogram to determine the impact of strong molecular interaction between the polymer molecules. For instance, the findings indicate that polymers’ aging can be experimented upon through polynomial assessments to determine the resistance properties of polymeric materials.

Lastly, this research reported that polymer performance can be improved through various approaches to shield the mechanical properties of polymeric materials from being destroyed by UV radiation. Notably, the results reveal that using additives can help stabilize polymers and, therefore, improve their performance against adverse environmental conditions like light, oxygen, and UV radiation that favor oxidation. As an illustration, the study discussed the potential use of carbon black as an additive that helps shield polymers from degradation. This substance gives polymers mechanical protection by absorbing UV radiation. The results also reiterate the potential for improving structural elements of polymers as a pathway towards enhancing their performance against UV radiation. The findings reveal that integrated waveguides can be used to enhance communication on the polymer’s structures. Other methods discovered in this study include the utilization of doping and redoping procedures to improve the effectiveness as well as dielectric features of polyaniline-coated polyester fabric. Additionally, enhancing the communication structures of polymeric materials can help relay information to those responsible for changes in mechanical conditions to enable them to take any corrective action to enhance their durability. These mechanisms provide a heightened shielding effect on polymeric materials by changing their surface resistivity and dielectric permittivity.

Overall, the study established that exposing polymeric materials to extreme environmental conditions, particularly humidity and UV radiations, accelerates the speed of polymer aging through thermal oxidation. Thus, in order to overcome the threat of accelerated aging, especially in industries such as transport and agriculture, where polymeric materials are used to enhance productivity and leverage other economic gains, practitioners must embrace additives and stabilizers. These materials can be mixed with polymers to help shield them from the adverse impacts of UV radiation on their aging processes.

## Figures and Tables

**Table 1:** Combinations of keywords with Boolean search strings used to form search terms for article identification.

Keywords	Boolean Operator	Search Term/Phrase
Polymer, degradation, aging, UV radiation, thermal conditions	OR	Polymer degradation OR aging under UV radiation Effect of solar radiation OR thermal conditions on polymer aging
Polymer, composite, electromagnetic, radiation, decomposition, aging, stabilization	AND	Polymer composites’ degradation under electromagnetic AND stabilization Polymer aging AND stabilization

**Table 2:** Examples of widely used polymers and their market share [183].

Polymer	Chemical Name	Market Share (%)
Polyvinylchloride	PVC	11.80
Polystyrene	PS	7.60
Polyethylene	PE	36.30
Polypropylene	PP	21
Polyethylene terephthalate	PET	10.20
Polyurethane	PU	8.2

## Data Availability

No data available.

## Abbreviations

UV	Ultraviolet rays
ABS-PC	Acrylonitrile Butadiene Styrene Polycarbonate
3D polymer	Three-dimensional polymer
(μ-POF)	Micro-polymer optical fibers
SBS	Styrene–butadiene–styrene
LDPE	Low-density polyethylene
UVA	Ultraviolet radiations with long wavelengths of between 400 and 320 nm
UVB	Ultraviolet radiations with a medium wavelength of between 320 and 290 nm
UVC	Short wavelength radiation with radiations of between 290 and 200 nm
PO	Polyolefin
CI	Carbonyl indexes
TiO_2_	Titanium dioxide
PLA	Polylactide
PISA	Porosity-Induced Sidechain Adsorption
PAF-1	A porous aromatic framework
PMMA	Poly(methyl methacrylate)
PANI	Polyaniline
PS	Polystyrene
PE	Polyethylene
CLSM	Confocal laser scanning microscopy
Tg	Transition temperature

## Appendix A. Summary of Literature Analysis

**Number**	**Authors**	**Title**	**Methodology**	**Findings**	**Conclusion/Recommendation**
[53]	Zhang et al.	Investigation of polymer aging mechanisms using molecular simulations: a review	Literature review	Polymer aging can be explained through the theory of reaction.	The theory of diffusion suggests that free permeating molecules react with polymer compounds, causing degradation
[54]	Liu and Li	Progress in study of polymer degradation behaviors and mechanisms in various environment conditions	A literature review study	Polymer aging differs from one environment to the other. A secondary bond change occurs during physical aging, although it is reversible.	Photo, thermal and chemical media influence polymer degradation
[55]	Scott	Initiation processes in polymer degradation.	A quantitative study was utilized	In thermal degradation of vinyl polymeric materials, free radicals are propagated through thermooxidative degradation.	Free radicals have a participatory role in polymer aging as they interact with oxidative agents
[56]	Day et al.	Degradation of contaminated plastics: A kinetic study	An experimental study focusing on four polymeric materials was implemented	The thermal degradation rate is even higher in polymers with metal contamination as that catalyzes their aging behavior.	Contamination increases the rate of polymer degradation
[57]	Troev et al.	Chemical degradation of polyurethanes. II. Degradation of microporous polyurethane elastomer by phosphoric acid esters	An experimental research design was implemented to test the degradation of microporous polyurethane elastome	phosphoric acid added to polyurethane was found to speed up degradation by breaking the free radicals, leading to the breaking up of the compound and making the trialkyl esters in this polymer.	The aging properties of polymers can be sped up the reactive agents that release free radicals
[58]	Barrer	Some properties of diffusion coefficients in polymers	A quantitative study using factor analysis and experimental observations was used	Through thermal fluctuation, fragments of polymers absorb energy, loosen and reshuffle themselves, making permeating molecules take new positions.	The study supports the theory of polymer reaction as free radicals react with permeating molecules
[59]	Arya et al.	Glassy Polymers—diffusion, sorption, ageing and applications	Quantitative research using diffusion models to explain polymer aging	Minute molecules of polymers diffuse with changes in temperature and concentrations.	Changes in the external conditions lead to reactions with polymer molecules
[60]	Enomoto et al.	The role of hydroperoxides as a precursor in the radiation-induced graft polymerization of methyl methacrylate to ultra-high-molecular-weight polyethylene	A quantitative research method using grafting technique was implemented	Solvents increase the free volumes of polymers as diffusion progresses through the free-volume chains.	Hydroperoxides can be used as solvents to increase the diffusion of polymer molecules through free volumes
[61]	Ching et al.	Effects of high temperature and ultraviolet radiation on polymer composites	A literature review methodology was used	High thermal conditions and UV radiations weaken the mechanical properties of polymer composites.	Exposing polymers to extreme thermal conditions causes them to age faster
[62]	Henry et al.	White LED induced photooxidation aging of epoxy/carbon fibre reinforced polymer and its implications for cleanroom cleanliness and contamination control	An experimental research design was implemented	Polymer degradation is caused by a photooxidation process of a UV source with up to 500 nm irradiation wavelengths. It affects the first microns of an irradiated surface.	Photooxidation polymer resins should be limited and avoided in some circumstances to deter the risks of aging
[63]	Lu et al.	Particle removal mechanisms in synergistic aging of polymers and glass-reinforced polymer composites under combined UV and water	Quantitative research was conducted through hydrodynamic analysis	The extra total mass that is unoccupied causes mass transport within the polymer structure.	The hydrothermal environments speed up the process of polymer degradation
[64]	Amza et al.	Effect of UV-C radiation on 3D-printed ABS-PC polymers	A quantitative study using experimentation to determine the impact of UV radiation on 3D polymer samples	3D polymers are more stable and withstand UV radiationbetter than other polymer types. 3D polymers can withstand UV radiations of wavelengths 200 nm to 280, making them resistant to UV aging.	3D polymers are more stable when exposed to aging conditions than normal polymer composites
[65]	Amza et al.	Aging of 3D-printed polymers under sterilizing UV-C radiation	Experimental research was conducted to determine the stability of normal 3D PLA when exposed to UV aging	3D-printed parts of a polycarbonate and acrylonitrile butadiene styrene polymer (ABS-PC) exposed to UV radiation experience minimal degradation.	3D parts can be used to sterilize polymers
[66]	Afshar and Mihut	Enhancing durability of 3D-printed polymer structures by metallization	Experimental study that used ABS specimens that were 3D-printed using fused deposition modeling (FDM) technique as well as metalized through DC magnetron sputtering	3D polymers lose their stiffness and retain their tensile strength following exposure.	3D polymers are more durable
[67]	Amza et al.	Accelerated aging effect on mechanical properties of common 3D-printing polymers	Quantitative study that entailed conducting scanning electron microscopy (SEM) fractographic analysis	3D parts did not lose stiffness after the UV-B exposure. Creep behavior was closely linked to the decline in mechanical properties	UV-B exposure substantially reduces 3D polymers’ tensile strength
[68]	Franco et al.	Accelerated ultraviolet aging of a PET nonwoven geotextile and thermoanalytical evaluation	The study used an experimental design to test the thermal behavior of commercial nonwoven polyester geotextiles exposed to accelerated UV aging	UV aging induced mechanical deterioration on polyester fibers.	Additives can be used to improve the stability of polymers during manufacturing
[69]	D’Urso et al.	PLA melt stabilization by high-surface-area graphite and carbon black	Experimental study using rheological measurements and gel permeation chromatography (GPC)	Carbon black can be used in small amounts to improve the resistance of polymers against aging.	Additives can improve the resistivity of plastic materials against thermal-induced aging
[70]	Dubey et al.	Carbon black-filled PE/PP/EPDM blends: phase-selective localization of carbon black and EPDM-induced phase stabilization	An experimental study using the melt compounding of PE, PP, ethylene propylene diene monomer (EPDM) as well as CB in different proportions	This additive helps absorb UV radiation and, therefore, protects the polymer component against degradation.	The percolation of carbon black in various phases emphasizes its role in reducing polymer degradation
[71]	CABOT.	Carbon blacks for protection of plastics exposed to ultraviolet light	Literature review study	Small particles of carbon black are more effective in shielding polymers from UV radiation.	Carbon black is effective in stabilizing polyethylene against UV radiation
[72]	Hachicha and Overmeyer	Investigation on aging of metallic surface-integrated micro-POFs	Quantitative research using optimal communication to test the mechanical features of polymers with micro-POFs	Improving the communication structures of polymers through integrated waveguides is also suggested.	Such novel methods provide another suitable approach for improving the properties of polymer aging
[73]	Hoghoghifard et al.	Improving EMI shielding effectiveness and dielectric properties of polyaniline-coated polyester fabric by effective doping and redoping procedures	Experimentation using X-band frequency range analysis	Doping and redoping procedures can improve the efficacy and dielectric features of polymer fabrics. Double and triple layers have attenuated shielding effects due to the absorption mechanism.	The redoping process compensates for the increase in surface resistivity as polymer samples are washed
[74]	Egghe et al.	Comparative study of the aging behavior of plasma-activated hexamethyldisiloxane-based plasma polymers and silicone elastomer thin films	A quantitative comparative study was conducted through experimentation	Enhancing the mobility of oxidized short-chain fragments has been reported as effective in polymer stability.	Some aging processes get reduced in crosslinked plasma activated films
[75]	Vasylius et al.	Degradation of mechanical properties of A-PET films after UV aging.	The study conducted an experiment with A-PET films exposed to UV aging for varying lengths of time	Extensive exposure can, however, make A-PET films lose tensile strength.	The duration of exposure determines the degree of deterioration
[76]	He et al.	Profile monitoring-based quality control method for fused deposition modeling process.	Quantitative research based on a non-contact measurement method was implemented, coupled with a statistical process control method	3D printing can utilize profile monitoring techniques to improve the quality of polymer performance.	The performance of polymeric materials in withstanding UV aging significantly differs from one material to the other
[77]	Mondal et al.	Effect of thermal–air aging treatment on mechanical properties and electromagnetic interference shielding effectiveness of low-cost nano-structured carbon-filled chlorinated polyethylene	A quantitative study using FESEM analysis was conducted	Low-cost CPE composites can be filled with VXC particles through a strategy of melting and mixing them to suppress EM pollution.	This method enhanced the interfacial polarization of the polymer, causing outstanding polymer–filler interaction
[78]	Lajić et al.	Accelerated and natural aging of offset prints covered with different varnishes.	Experiments of natural aging and accelerated aging were conducted	Polymers are used in manufacturing plastics across various industries, such as varnishes.	Polymeric materials have broad industrial uses
[79]	Cantero-Chinchilla et al.	Robust optimized design of 3D-printed elastic metastructures: A trade-off between complexity and vibration attenuation	An experimental study using a transfer matrix approach was conducted	3D-printed metastructures can be optimized strategically through the robust design of mechanical metamaterials.	Smaller variabilities in material properties impact the robustness of the metamaterials
[80]	Mantada et al.	Parameters influencing the precision of various 3D printing technologies	Experimental research comparing the precision of 3D printing technologies	3D printing precision can be improved through Fused Deposition Modeling (FDM), Stereolithography (SLA), Polyjet Matrix and Selective Laser Sintering (SLS).	With such high precision, these 3D printing technologies can be used across various industries
[81]	Lin et al.	Effects of ultraviolet aging on properties of wood flour–poly(lactic acid) 3D printing filaments	A comparative study using experimental research to determine polymer aging through color, tensile strength, scanning electron micrographs, as well as water absorption rate of polymer filaments	Polymers are also utilized in making PLA 3D printing filaments due to the aging resistance of these filaments.	Adding UV absorbent into polymer filaments improves their aging resistance
[82]	Sousa et al.	Mechanical properties of 3D printed mouthguards: Influence of layer height and device thickness	Experimental study focusing on the thickness and printing quality (layer height)	There was a decline in the tensile strength as specimen thickness increased, with the exception of TPU because of its highest level of deformation capacity.	Customizing mouthguards with thinner walls through additive processing techniques can hinder injurious outcomes
[83]	Ferreira et al.	Ageing effect on the low-velocity impact response of 3D printed continuous fiber reinforced composites	An experimental study using low-velocity impact tests across varying numbers of days was conducted	The initial elastic part of 3D-printed continuous fiber-reinforced composites slightly reduces as the polymer ages.	The severity of visual damage to the polymer specimens varied with age
[84]	Najvani et al.	Early-age strength and failure characteristics of 3D printable polymer concrete.	The study was based on a rheology analysis	The rheological data could be integrated into the Herschel–Bulkley model. The flow index for the polymer concrete decreased with time, while the static yield stress, thixotropy, as well as consistency were observed to increase.	Polymer concretes are also used in construction instead of cement
[85]	House et al.	Case report of asthma associated with 3D printing	Case study design	3D polymers, however useful in many industries, can cause asthmatic and other health challenges.	There is a need for further research to determine whether 3D polymers have health detriments
[86]	Bedi et al.	Reviewing some properties of polymer concrete	Secondary research methodology based on a literature review	Polymer concretes’ characteristics differ greatly based on their conditions of preparation.	Precast polymer concrete is being used to manufacture various products such as acid tanks, manholes, drains, and highway median barriers
[87]	Krčma et al.	Use of polymer concrete for large-scale 3D printing	The quantitative study used the deposition modeling process and computed tomography of polymer samples	The 3D-printed samples showed high levels of porosity.	The new polymer material is useful in 3D printing, with negligible to no degradation resulting from the process
[88]	Furet et al.	3D printing for construction based on a complex wall of polymer-foam and concrete	An experimental study was conducted	Polymer concretes are mixed with fillers, resins, and aggregates to provide a substitute binder for cement in additive manufacturing.	Additive manufacturing uses 3D printing to create foam and extrude the concrete with a robotic system to jointly form both the structure as well as thermal elements of the structure/building
[89]	Kozicki et al.	Measurement of the radiation dose and radiation isocenter of the truebeam accelerator using 3D polymer gel dosimeters from the VIPAR family with different chemical history	Experimental research using three-dimensional polymer gel dosimeters was conducted	N-vinylpyrrolidone acts as a stabilizing agent for dosimeters.	Ionized dosimeters are also products of 3D polymers
[90]	Chapiro	Radiation effects in polymers	Quantitative study	Ionizing radiation causes polymer aging.	The crosslinking of shaped polymers coupled with the curing of coatings comprise broader applications of polymeric materials
[91]	Lebedev et al.	Radiation aging of polymer composite materials.	Experimental study	Polymer composites exposed to UV radiation deteriorate due to the weakening of their mechanical properties.	Physicochemical as well as structural changes in polymer matrices are observable when these composites are exposed to UV aging
[92]	Davenas et al.	Stability of polymers under ionizing radiation: the many faces of radiation interactions with polymers	Experimental research	As the polymer interacts with UV radiations, its mechanical properties weaken, causing aging.	Polymer stability is challenged under ionization agents
[93]	Feldman and Zezin	The 13th International Symposium on Ionizing Radiation and Polymers (IRaP, 2018)	Conference publication with n = 100 contributors drawn from 20 countries	The ionizing impacts of radiation can, at times, cause polymer degradation.	Despite ionization’s impacts, polymers are crucial in making composites with thermoplastic and thermoset matrices
[94]	Hakamivala et al.	Parametric process optimization to improve the accuracy and mechanical properties of 3D printed parts	A quantitative study using parametric analysis	Adding interactive 3D features to the polymer can improve scaffolding.	Parametric analysis can be used to enhance 3D-printed polymers’ accuracy and mechanical properties
[95]	Sun et al.	Oxidation and polymer degradation characteristics of high viscosity modified asphalts under various aging environments	Experimental study	Polymer aging through exposure to oxidized environments ruins the stability of plastics.	Exposing polymer resins and high-viscosity modified asphalts to oxidized environments accelerates their aging mechanisms
[96]	Lin et al.	Unraveling the influence of fibers on aging susceptibility and performance of high content polymer modified asphalt mixtures.	Case study research methodology based on lignin and polyester polymers as representative samples. Tests on Cantabro loss, SCB strength, SCB fatigue and Hamburg Wheel-Tracking were performed	Exposure of polymers to aging sources reduces their lifespans in real-life use. However, polymers can improve their porosity if combined with fiber mixtures.	High-Content SBS Polymer-Modified Bitumen (HCPMB) has excellent features in its original state that mask the enhancement impact of fibers
[97]	Makki et al.	Micromechanical modeling of the visco-hyperelastic-viscoplastic behavior and fracture of aged semicrystalline polymers.	Experimental study based on modeling analysis	The aspect of polymer degradation affects the longevity of high-viscosity modified asphalt.	Polymer aging accelerates the period within which polymeric materials have a useful economic life
[98]	Desidery and Lanotte	Identification of undisclosed modifiers and their effects on chemical, thermal, and microstructural properties of unaged and aged industrial high polymer-modified asphalt binders	Experimental study	The aging processes of polymeric materials affect the properties of the base bitumen, undisclosed crystalline modifiers, and SBS.	Modifiers perform better when the base characteristics of polymers have not been altered
[99]	Hu et al.	Performance study on anti-weather aging combinations for high-content polymer modified asphalt and comparison by improved multi-scale mathematical TOPSIS method	Experimental research based on TOPSIS calculations	Unique combinations of novel materials for light absorption and antioxidation, as well as those for shielding light, can provide a shielding effect against the aging of asphalt polymers.	Combining these materials appropriately can equip polymers with anti-aging characteristics
[100]	Goncalves Bardi et al.	Behavior of UV-cured print inks on LDPE and PBAT/TPS blend substrates during curing, postcuring, and accelerated degradation	Experimental research method	Polymer reactions generate curative blend substrates.	Using print inks improves the stability of polymer substrates and polymer composites against UV aging
[101]	Wang et al.	Waterborne polyurethane composite coatings as UV-light converter and directional infrared light barrier for light and heat management in greenhouse	Experimental	Composite coatings on the greenhouse plastics increased pepper yield by 35%, while each fruit added 8 g in weight.	These coatings can be used to increase agricultural yield
[102]	Vijayalakshmi and Baek	Conversion of UV light to dazzling reddish orange light with robust color purity for plant growth in biocompatible glasses	Experimental design	Adding Gd3+ to red emissions of Eu3+ optimized glass productivity in agricultural production.	There is a profound applicability of plastics in agriculture since they convert UV light to the needed conditions for agricultural production
[103]	Al-Helal et al.	Effect of shape, orientation and aging of a plastic greenhouse cover on the degradation rate of the optical properties in arid climates	Experimental research was conducted on four greenhouses with two shapes	The shape and orientation of greenhouses with small sizes did not significantly impact the rate of degradation of the cover’s optical properties. The four corners examined had reduced global solar radiation transmittance (T) by 27–31% after one year of exposure relative to the new film.	These plastics can also degrade if exposed to intense conditions
[104]	Dehbi et al.	Degradation assessment of LDPE multilayer films used as a greenhouse cover: Natural and artificial aging impacts	Experimental research	The rate of aging is influenced by surface location and the orientation of the cover.	The degree of degradation differs based on the surface location of the LDPE multilayer films
[105]	Bonyadinejad et al.	Investigating the sustainability of agricultural plastic products, combined influence of polymer characteristics and environmental conditions on microplastics aging	Experimental	Low-molecular weight-microplastics have less photostability.	Covering LDPE with soil made them experience limited photo-degradation
[106]	Rabaev et al.	Long term aging of LLDPE based multi-layer film by exposure to light hydrocarbons.	Experimental study	Photo-degradation is particularly reported for LDPE green-house plastics due to their interaction with UV radiation.	The longest container stayed for 10 years before degradation
[107]	Ji et al.	Composition identification and UV-C irradiation growth inhibition effect of green shading on the greenhouse cover	Experimental study	Shading has a shielding effect on the inhibition of UV-C irradiation.	Shading through the correct anti-aging components can increase the longevity of LDPE covers
[108]	Tuasikal et al.	Influence of natural and accelerated weathering on the mechanical properties of low-density polyethylene films	Experimental study	Adding anti-oxidants helps reduce the aging effect of UV radiation on LPDE covers.	Natural and accelerated aging can be inhibited through anti-oxidants
[109]	Amin et al.	Natural weather ageing of the low-density poly-ethylene: Effect of polystarch N	Experimental study	The degree of photo-degradation is affected by the disposition of soil particles.	If the soil particles are correctly dispositioned, then polymers used for agriculture can have an extended lifespan
[110]	Picuno	Innovative material and improved technical design for a sustainable exploitation of agricultural plastic film	Literature review methodology	Improving the technical design of agricultural plastics can elevate their antiaging performance.	Agricultural plastic films can be recycled and reused to prevent externalities
[111]	Djakhdane et al.	The effect of sand wind, temperature and exposure time on tri-layer polyethylene film used as greenhouse roof	Experimental research	PE film is highly degradable after exposure to extreme heat and sand wind.	Sand wind and high temperatures are factors that facilitate polymer degradation
[112]	Dehbi et al.	Degradation of thermomechanical performance and lifetime estimation of multilayer greenhouse polyethylene films under simulated climatic conditions	Experimental study	The highest level of degradation was noted for plastics exposed to the combined impacts of temperature and UV radiation.	Thermal–oxidative conditions significantly deteriorate the performance and functionality of plastics
[113]	Bonhomme et al.	Environmental biodegradation of polyethylene	Experimental research	Microorganisms and oxidative products facilitate polymer aging.	Extreme temperatures and humidity accelerate polymer aging
[114]	Garnai Hirsch et al.	Characterization of surface phenomena: probing early stage degradation of low-density polyethylene films.	Experimental study	The contact angles of the plastics were sensitive to nanoscale surface roughening as well as composition changes.	At early aging there is poor sensitivity to the deterioration of mechanical properties
[115]	Hirsch et al.	Correlating chemical and physical changes of photo-oxidized low-density polyethylene to the activation energy of water release.	Experimental study	Release of water correlated with accelerated LDPE photo-oxidation.	Exposure to high humidity lowers the surface changes on the plastics
[116]	Picuno et al.	Agrochemical contamination and ageing effects on greenhouse plastic film for recycling.	Experimental research	Agrochemicals increased the aging properties of greenhouse plastics.	Agrochemicals reduce the lifespan of agricultural plastics
[117]	Schettini et al.	Interaction between agrochemical contaminants and UV stabilizers for greenhouse EVA plastic films	Experimental research	The interaction of agrochemicals with greenhouse plastics contaminates them.	Agrochemicals also speed up the degradation of agricultural plastics
[118]	Lamnatou and Chemisana	Solar radiation manipulations and their role in greenhouse claddings: Fresnel lenses, NIR-and UV-blocking materials.	Experimental research	UV radiation is very destructive for greenhouse plastics. However, using UV-blocking materials reduces the adverse impacts of solar radiation.	Solar radiations weaken the mechanical strength of agricultural plastics
[119]	Hamzah et al.	Surface chemistry changes and microstructure evaluation of low-density nanocluster polyethylene under natural weathering: A spectroscopic investigation	Experimental research	Natural weathering gradually degrades the mechanical properties of polyethylene.	Natural weathering reacts with the polymer surface; thus, the plastics’ mechanical properties are weakened, and their useful lives are reduced
[120]	Dehbi et al.	Artificial ageing of tri-layer polyethylene film used as greenhouse cover under the effect of the temperature and the UV-A simultaneously.	Experimental research	Carboxyl groups begin to degrade after extensive exposure to UV radiation.	Artificial aging shows how the degradation parameters in the natural environment cause polymer aging
[121]	Dehbi et al.	Impact of degradation of polyethylene films under simulated climatic conditions on their mechanical behavior and thermal stability and lifetime	Quantitative study based on simulations	Climatic conditions significantly cause the deterioration of agricultural films.	The film’s mechanical properties and material structure were significantly altered when the experiment was performed in a simulated environment
[122]	Lycoskoufis et al.	Ultraviolet radiation management in greenhouse to improve red lettuce quality and yield	The study employed two experiments to determine how UV light improved quality and productivity	The study determined that using supplemental UV lighting of dosage 425 kJ m^−2^ d^−1^ for 10 days prior to harvesting produced red lettuces of similar quality as those grown in UV-open greenhouses. Lettuce yield increased by 30% without any adverse effects.	UV light is converted by greenhouse plastics into favorable light conditions for maximizing productivity
[123]	Zou et al.	. Solar spectrum management and radiative cooling film for sustainable greenhouse production in hot climates	Literature review	Greenhouse management provides a viable solution to tackling the global food crisis	The UV radiation entering greenhouses must be managed within 400–700 nm to avoid degrading the agricultural plastics used to promote crop production in hot climates
[124]	Elanmugilan et al.	Natural weather aging of low-density polyethylene: effect of prodegradant additive	A quantitative study using rheological and Fourier transform infrared spectroscopy (FTIR) and scanning electron microscope (SEM) was implemented	In natural weathering processes, degradation of LDPE occurs as a result of chain scission and crosslinking.	Electromagnetic radiation of 300 and 400 nm wavelengths causes the natural aging of LDPE plastics. Adding additives elongates the lifespan of these plastic materials
[125]	Siti et al.	Mechanical properties of UV irradiated bio polymer thin films doped with titanium dioxide	The study used an experimental research design whereby film samples were irradiated in a UV-accelerated weatherometer at temperatures of 50 °C with varying durations of exposure	The study discovered that as the wavelengths increased, more free radicals were released from the polymer structure.	Neat BPF coupled with BPF doped with 10% TiO2 and then subjected to UV radiation showed higher ductility than the unexposed BPF. The longer BPF was exposed to UV light, the more it had a systematic increment in tensile strength because of the heightened crosslink between isocyanate as well as the hydroxyl group
[126]	Katsoulas et al.	Plant responses to UV blocking greenhouse covering materials: A review	The study conducted a literature review based on a sample of representative studies to show the impact of UV-blocking materials on different crops’ agronomic factors	The results show that UV shielding materials had a positive impact on the physiological functions of different plants. These functions included the transpiration rate as well as photosynthesis, thus contributing to plants’ growth characteristics.	Anthocyanins and total phenolics are used to block UV radiation in greenhouses
[127]	Al-Salem et al.	Study of the degradation profile for virgin linear low-density polyethylene (LLDPE) and polyolefin (PO) plastic waste blends	The properties of LLDPE and PO were studied experimentally. The techniques used included thermogravimetry, differential scanning calorimetry (DSC), as well as infrared spectroscopy and scanning electron microscopy (SEM).	The polymers underwent crystallinity because of losing weight to weathering, leading to a change in the crystal size.	Polymers exhibit immiscibility as well as polydispersity when contextualized within the blending matrix as a consequence of chain scission and oxidation following UV exposure
[128]	Feldman	Polymer weathering: photo-oxidation	The study reviews the literature on polymer weathering	Weathering of polymers is severe since it merges photophysical and photochemical impacts of UV radiation with oxidative as well as hydrolytic impacts of the outdoor environment.	Photooxidation of polymers results from the degradation of their mechanical properties due to outdoor factors
[129]	Andrady et al.	Consequences of stratospheric ozone depletion and climate change on the use of materials	A literature review was conducted to describe technical advances in the techniques of stabilization and degradation of polymers	Weather-induced degradation of plastic and wood materials reduces their service life.	The interaction of polymers with UV-B causes them to have shorter lifespans
[130]	Chen et al.	A flexible UV–Vis–NIR photodetector based on a perovskite/conjugated-polymer composite.	Experimental research design	The conjugated polymer composite developed had outstanding mechanical flexibility coupled with improved environmental stability.	The paper inspired the development of diverse perovskite-based polymer composite systems that deliver superior performance at low costs and elevated-performance flexible broadband photodetectors
[131]	Al-Salem et al.	Effect of die head temperature at compounding stage on the degradation of linear low density polyethylene/plastic film waste blends after accelerated weathering	Experimental study that tested accelerated weathering of LLDPE	It was observed that exceeding the melting point of polymeric blends did not always lead to their synergistic behavior.	Once subjected to UV exposure under the conditions of weathering, polyolefin polymers lose their amorphousness due to the impact of plastic waste components
[132]	Hu et al.	Understanding the aging depth gradient distribution of high viscosity modified asphalt under the effect of solar radiation and diffuse oxygen.	An experimental study on the aging of high-viscosity modified asphalt (HVMA) was performed	The impact of UV radiation deterioration diffused from the surface of the asphalt to the asphalt inside in a gradual manner.	As the intensity of the solar radiation increased and aging time advanced, the aging depth of HVMA also increased continuously
[133]	Ni et al.	Degradation characteristics of SBS polymer and its contribution to weathering aging of modified asphalt	The study used shear rheology experiments (DSR) to determine the aging behaviors of SBS polymers	The findings illustrate that the deterioration pattern of weathering SBS obeys a single function of exponential decay function.	The effective content of the SBS decreased to 66.7%, 50%, as well as 33.3% post-accelerated weathering for 5.8, 10.5 and 14.5 h, respectively.
[134]	Garg et al.	Melamine–Formaldehyde Polymer-Based Nanocomposite for Sunlight-Driven Photo-degradation of Multiple Dyes and Their Mixture.	The study applied a comparative quantitative study methodology through nuclear magnetic resonance (NMR) analysis of polymer composites	A breakdown of organic dyes’ aromatic framework through the MFP-CdS in sunlight was observed.	Organic dyes undergoing photodegradation under UV radiation due to the substantial involvement of superoxide radicals and their holes.
[135]	Ávila-López et al.	Photo-degradation of air and water contaminants using 3D-printed TiO_2_ nanoparticle scaffolds	Experimental research	The addition of water and air contaminated the polymer, thus accelerating its aging process.	The susceptibility of polymers to aging stems from the weak carbon bonds that UV radiation breaks
[136]	Cacuro et al.	Demonstration of polymer photo-degradation using a simple apparatus	Quantitative research based on simple tests was conducted in four classes with n = 25 students	Photodegradation can use recent technology to functionalize non-polar polymer surfaces.	Exposure of polymer surfaces to photodegrading elements causes them to deteriorate in terms of their mechanical properties
[137]	Garg et al.	Ag_3_PO_4_ nanoparticles-decorated melamine–formaldehyde polymer nanocomposite as a catalyst for the photo-degradation of bisphenol a and its antibacterial activity	Experimental research design	The polymer nanocomposites fractured their molecular weight upon photodegradation and released free radicals.	The nanocomposite showed effective antibacterial activity
[138]	Lei et al.	Aerosol acidity sensing via polymer degradation	Experimental research based on pH measurement	As the size of the particle decreased, there was an increase in polymer degradation, showing a rise in aerosol acidity at smaller diameters of the particle.	This new approach can be used in the determination of particle acidity
[139]	Palkar and Kuksenok	Controlling degradation and erosion of polymer networks: insights from mesoscale modeling	Experimental study utilizing reverse gel point measurement	This approach was able to prevent polymer percolation within the network	Nanoscale modeling can help prevent polymer degradation
[140]	Cheng et al.	Hyper cross-linked additives that impede aging and enhance permeability in thin polyacetylene films for organic solvent nano-filtration	The experimental method was conducted through organic solvent nanofiltration	The study was able to develop scalable, thin film nanocomposite membranes that were supported on polymer substrates that resist physical aging while at the same time having high permeability rates to alcohols.	Thus, developing resistant membranes in polymers is can enhance the durability of polymer composites
[141]	Fraga Dominguez et al.	Unravelling the photo-degradation mechanisms of a low bandgap polymer by combining experimental and modeling approaches	Experimental research	The method was effective in determining the photodegradation properties of polymers.	Combining experimental methods with modeling offers a superior approach to polymer aging
[142]	Rivas Aiello et al.	Magnetic nanoparticle–polymer composites loaded with hydrophobic sensitizers for photo-degradation of azoic dyes	Experimental study design	There was a 76% efficiency of the dye decomposition at the pH value of 6.	Similar approaches could be applied in encapsulating hydrophobic photosensitizers
[143]	Tian et al.	Amphiphilic polymer micellar disruption based on main-chain photo-degradation	Experimental research	The N–O photocleavage can accelerate the release of DOX in aqueous media.	There is a rapid degradation of polymers in hydrophobic environments
[144]	Xiu et al.	Simultaneously improving toughness and UV-resistance of polylactide/titanium dioxide nanocomposites by adding poly (ether) urethane	Experimental research	Adding TiO_2_ to PLA can enhance its resistance to aging.	Poly(ether) urethane can be added to polymer surfaces to improve their UV resistance
[145]	Zhou et al.	Photocatalytic degradation by TiO_2_-conjugated/coordination polymer heterojunction: Preparation, mechanisms, and prospects.	Experimental study	Conjugated polymer with TiO_2_ exhibited improved resistivity to photocatalytic degradation.	The addition of TiO_2_ to conjugated polymers improves their anti-aging properties
[146]	Smith et al.	Control of physical aging in super-glassy polymer mixed matrix membranes.	Experimental study	PISA can enhance polymer stability against physical aging.	The addition of PISA can improve the resistance of super-glassy polymers against physical deterioration
[147]	El-Hiti et al.	Modifications of polymers through the addition of ultraviolet absorbers to reduce the aging effect of accelerated and natural irradiation	Experimental study	The absorbent material applied to the polymer surface helped modify its ability to withstand UV-induced weathering.	Absorbers are suggested for use to enhance polymer stabilization
[148]	Zemke et al.	Applications of the Tachiya fluorescence quenching model to describe the kinetics of solid-state polymer photo-degradation	Experimental study	The Tachiya model provided improved fits to the experimental kinetics data when considering the short reaction time.	Polymers with metal–metal bonds are also helpful in circumventing photodegradation
[149]	Auras et al.	Poly (lactic acid): synthesis, structures, properties, processing, applications, and end of life,	A book series providing a series of experimental and literature reviews	Eco-friendly PLA polymers have numerous industrial uses ranging from packaging to medical implants as well as wastewater treatment.	Polymeric materials require end-of-life management to ensure that they are used properly in their diverse use functions
[150]	Wallnöfer-Ogris et al.	Main degradation mechanisms of polymer electrolyte membrane fuel cell stacks–Mechanisms, influencing factors, consequences, and mitigation strategies	Review of literature	Operating conditions cause voltage decay in fuel cells as the polymer surfaces degrade.	Initial degradation influences further deterioration of the fuel cell
[151]	Karlsson and Albertsson	Techniques and mechanisms of polymer degradation	Literature review	Exposing polymer materials to complex outdoor environments makes them susceptible to decay.	A combination of oxidative agents causes polymer degradation
[152]	He et al.	Polymer degradation: category, mechanism and development prospect	Literature review	Degradation methods such as photo-, oxidative, catalytic, and biodegradation can help address the problem of white pollution.	An understanding of polymer degradation methods provides integral details on how to manage plastics
[153]	Ray and Cooney	Thermal degradation of polymer and polymer composites	Literature	Thermal environments accelerate the process of polymer decomposition.	With the increase in the commercial use of polymers, it is crucial to determine effective aging mechanisms for proper end-of-life management
[154]	La Mantia et al.	Degradation of polymer blends: A brief review	A literature review	Degradability and durability define the usefulness of polymeric materials.	The generation of free radicals by polymers once exposed to thermal and oxidative environments causes polymer aging
[155]	Ricci et al.	Linear stress relaxation and probe reorientation: comparison of the segmental dynamics of two glassy polymers during physical aging	Experimental research	The study used optical probe reorientation as a method for determining polymer aging.	Various methods can be applied during experimentation
[156]	Hebert and Ediger	Reversing strain deformation probes mechanisms for enhanced segmental mobility of polymer glasses	Experimental study	Enhanced segmental mobility can be applied to reverse deformations in polymers.	After reversal, deformations reaching 60% of the yield strain were observed
[157]	Hodgson et al.	Studying thermally induced chemical and physical transformations in common synthetic polymers: A laboratory project.	Laboratory experimentation	Polymer analysis and polymer characterization are useful in indicating the changes in aged polymers.	Common synthetic fibers experience both physical and chemical aging due to thermal influence
[158]	Han et al.	Metalloporphyrin-based DA type conjugated organic polymer nanotube for efficient photocatalytic degradation	Experimental study	The transfer and removal of photo-induced carriers were greatly improved through metal-to-ligand charge transfer (MLCT)	New methods can provide valuable ways through which photocatalytic activities can be enhanced in polymer aging experiments
[159]	Dinoop lal et al.	Accelerated photo-degradation of polystyrene by TiO_2_-polyaniline photocatalyst under UV radiation	Experimental study	UV radiation caused the faster degradation of the polymer.	Nano-TiO_2_ and PANI are also used to establish the aging features of polymers and their composites.
[160]	Mohammed et al.	Protection of poly(vinyl chloride) films against photo-degradation using various valsartan tin complexes	Experimental study	Lifespan elongation of polymers uses field-emission scanning electron microscopy.	Non-desirable transformations were lower in films with tin complexes compared to blank polymeric films
[161]	Ding et al.	Investigation of the thermal degradation of SBS polymer in long-term aged asphalt binder using confocal laser scanning microscopy (CLSM)	Experimental research	Confocal laser scanning microscopy (CLSM) characterized the morphological alterations of the SBS polymer.	Increasing the aging temperature accelerated the degradation of the SBS polymer while maintaining the same rheological level
[162]	Verney et al.	Melt viscoelastic assessment of poly (lactic acid) composting: influence of UV ageing.	Experimental research	Melt viscoelastic assessment helped establish the influence of UV aging on the polymer.	When this method is applied in polymer aging the specimens undergo deep molecular evolutions
[163]	Lesiak et al.	UV sensor based on fiber Bragg grating covered with graphene oxide embedded in composite materials.	Experimental study	Using UV sensors cause epoxy resins to undergo degradation.	UV lamps with wavelengths 290–400 nm also experimentally determine how the epoxy matrices of polymers age under UV exposure
[164]	Zhang et al.	3D printed lignin/polymer composite with enhanced mechanical and anti-thermal-aging performance	The study applied an experimental research design to perform the demethylation of hardwood organosolv lignin	3D-printed lignin had better aging performance against mechanical and thermal aging	Modifying lignin through the phenolic enhancement of its structure improved its interfacial adhesion.
[165]	Glaskova-Kuzmina et al.	Durability of biodegradable polymer nanocomposites	Literature review	Incorporating nanofillers into biodegradable polymers could attenuate the loss of mechanical properties as well as improve durability.	It is crucial to note that, at times, nanofillers can contribute to substantial polymer degradation
[166]	Biale et al.	A systematic study on the degradation products generated from artificially aged microplastics	An experimental study was conducted to artificially age five microplastics to understand their degradation behavior. The experiments used absorption spectroscopy to monitor how polymers’ physical and chemical properties are transformed after exposure	The photo-aged microplastics had remained with about 18 wt. % of extractable and low-molecular-weight fraction. The remaining fraction differed from one polymer to the other depending on the type.	There was a marked decline in the mean molecular weight of the polymer chains of PP, while PS had crosslinking
[167]	Suraci et al.	Degradation assessment of polyethylene-based material through electrical and chemical-physical analyses	The study conducted experimental research whereby two LDPE flat specimens were subjected to thermal aging under different temperature conditions. The temperatures applied were 90 °C and 110 °C for the two polymers	The mechanisms of degradation were found to be deeply affected by the aging temperatures used.	There is a possibility of performing polymer aging development via non-destructive techniques that use electricity
[168]	Karimi et al.	A review on graphene’s light stabilizing effects for reduced photo-degradation of polymers	The paper reviewed existing knowledge about graphene	Using graphene, which is the latest member of the carbon group of metals, provides higher efficiency in increasing the shielding effect of polymers against photo-degradation. This superior performance of graphene is even possible at low loads of 1 wt. % or less. The protective role of graphene entails several complementary mechanisms that are aligned with the unique geometry as well as chemistry of the metal.	The findings showed that graphene’s free radical scavenging impact partially comes from the functional hydroxyl groups on its surfaces. Graphene also acts as a nucleating agent and can, therefore, enhance polymeric materials’ photostability
[169]	Urso et al.	Breaking polymer chains with self-propelled light-controlled navigable hematite microrobots	Experimental research design was applied	The method was effective in degrading polymers with high molecular weights through matrix-assisted laser desorption.	The effectiveness of the method was attributed to the microrobots’ active motion, improved capture of polymer chains through electrostatics and better charge separation
[170]	Giron and Celina	High temperature polymer degradation: Rapid IR flow-through method for volatile quantification.	The study used a quantitative research methodology	The rapid mid-IR gas analytical technique developed was useful in quantifying the volatiles contained in minute ampoules following exposure to the aging process.	The changes in the polymers’ mean molecular weights can be used to monitor changes
[171]	Plota and Masek	Lifetime prediction methods for degradable polymeric materials—A short review	The paper utilized a literature review methodology	The environmental factors causing the degradation of polymers are humidity and sunlight, which create thermal oxidative conditions. The polymers’ aging mechanisms can be predicted through lifetime analysis methods like the Arrhenius model and time–temperature superposition.	Accelerated aging tests and data extrapolation from induced thermal aging can determine the shelf life of polymers
[172]	Hebner and Maurer-Jones	Characterizing microplastic size and morphology of photodegraded polymers placed in simulated moving water conditions	An experimental research design was applied	When the polymers were irradiated for 1, 2, and 3 days, the results showed that an extensive period of irradiation correlated with the formation of more microplastics by the polypropylene films.	Thus, the changes in the polymer’s microplastics correlated with alterations in the glass transition temperatures. Irradiating the polymer at 300 nm generated fewer microplastics as a result of slower phototransformation kinetics.
[173]	Rånby	Photo-degradation and photo-oxidation of synthetic polymers	An exploratory study that examined the process of polymer aging through photooxidation	UV radiation damages polymer networks, which is determined through crosslinking.	Electron spectroscopy, infrared, and electron spin resonance spectroscopy can conduct chemical analyses of photoinitiated reactions
[174]	Kyrikou et al.	Analysis of photo-chemical degradation behavior of polyethylene mulching film with pro-oxidants	An experimental study was conducted	Declined weight of the polymer molecule and production of free radicals show that changes have occurred.	The combined impact of critical factors of pro-oxidants on the predefined photochemical degradation of LLDPE films is significant.
[175]	Briassoulis et al.	Combined effect of UVA radiation and agrochemicals on the durability of agricultural multilayer films	The study involved an experimental design for multi-layered films of 180–200 μm thick	Combining agrochemicals with UV radiation leads to the early failure of barring layers, which compromises the longevity of the whole polymer film	The study recommended the elimination of barrier layers in agricultural multilayer films to enhance their durability
[176]	Maraveas	Environmental sustainability of greenhouse covering materials	Literature review article	Polymeric materials can be recycled through mechanical as well as chemical means, closed-loop cycling and the polymerization of biodegradable feedstock.	Plastic films that have gone past the consumption phase do not have the same optical or energy characteristics as virgin polymers. The combined advantages of diverse polymeric materials underscore that they can be embraced on a large scale in the long run.
[177]	Maraveas et al.	Sustainable greenhouse covering materials with nano- and micro-particle additives for enhanced radiometric and thermal properties and performance	Literature review	Future innovations on the macro and micro synthesis of nanomaterials can provide an improvement in using them for greenhouse coverings.	These materials are, however, highly sensitive to external climatic as well as meteorological conditions
[178]	Maraveas et al.	Smart and solar greenhouse covers: Recent developments and future perspectives	Literature review	Greenhouses embedded with transparent polymers as roofs embrace sustainable approaches whereby the energy generated is fully renewable as well as economical.	The new use of polymer films that have tailored light absorbance as well as emission features regulates solar radiation. These materials also help regulate the internal and external temperatures of greenhouse agricultural projects
[179]	Zhong and Zhang	3D printing geopolymers: a review.	Literature review	3D printing technology, premised on the concept of additive manufacturing, fosters increased productivity of construction works	Geopolymers’ printability can potentially be optimized by blending fly ash, silica fume, solid activator, and river sand.
[180]	Rapisarda et al.	Photo-oxidative and soil burial degradation of irrigation tubes based on biodegradable polymer blends.	Experimental research design	Elevated temperatures or UV-induced aging led to the deterioration of the Bio-Flex^®^-based irrigation tubes in soil.	UV radiation sources cause the release of free radicals and a reduction in the molecular weight of polymers
[181]	Zaaba and Jaafar	A review on degradation mechanisms of polylactic acid: Hydrolytic, photodegradative, microbial, and enzymatic degradation.	Literature review	Other tests on degradation mechanisms use pyrolysis to determine how main chain polymer scission and polymer molecules unzip.	PLA has broader industrial applications, ranging from packaging to agriculture, textile, and biomedical uses, among others
[182]	Maléchaux et al.	Influence of gamma irradiation on electric cables models: study of additive effects by mid-infrared spectroscopy	Experimental research design	Models of insulation using crosslinked polyethylene (XLPE) and structural modification are discussed in relation to the impacts of additives. The results show the impacts of aging based on the dose rate as well as material formulation.	A low dose rate was found to cause elevated polymer degradation compared to a high dose rate
[183]	Ali et al.	Degradation of conventional plastic wastes in the environment: a review on the current status of knowledge and future perspectives of disposal	Literature review	Exposure of conventional plastics to the environment weakens their mechanical properties.	Plastic degradation can be improved by merging diverse degradation techniques. However, plastic degradation needs further research to establish biodegradation processes
[184]	La Mantia et al.	Comparative investigation on the soil burial degradation behavior of polymer films for agriculture before and after photo-oxidation.	A causal comparative study using experimentation was conducted	Biodegradable polymer films in soil experienced more degradation than PE-based polymers. UV irradiation increases polymers’ surface wettability coupled with its disintegration in the soil.	Photooxidation caused a reduction in the polymers’ molar mass as well as their hydrophilic end groups. This aspect increases the loss of weight and erosion of the surface by the polymer samples
[185]	Bhandari et al.	Degradation of fundamental polymers/plastics used in daily life: a review.	Literature review	Bio-based polymers degrade comparatively faster into fragments that are environmentally compatible than petro-based polymers.	It is crucial to produce bio-based polymers instead of petro-based ones to provide a sustainable solution that addresses plastic-related environmental concerns around the globe
[186]	Yu et al.	Impact of ultraviolet radiation on the aging properties of SBS-modified asphalt binders	This experimental study conducted 16 different tests to determine the impact	Consistent exposure to UV radiation increased the shrinkage stress in the polymer samples, making their surfaces crack.	The lab conditions mirror the usual natural conditions that cause polymer aging
[187]	Kaczmarek et al.	Effect of short wavelength UV-irradiation on ageing of polypropylene/cellulose compositions	Experimental study	As the epoxy resins are exposed to thermal extremities, with temperatures surpassing the glass transition temperatures, they age.	Reflectance infrared spectroscopy (ATR-FTIR) can be merged with SEM to provide a faster estimation of changes occurring in polymers during ecological degradation
[188]	Vašíˇcek et al.	Degradation of polylactic acid polymer and biocomposites exposed to controlled climatic ageing: mechanical and thermal properties and structure	Experimental research	Tensile and flexural tests showed that the biocomposites weakened their mechanical properties after exposure to thermal conditions due to the fragmentation of their chemical structure.	The fragmentation of biocomposites’ chemical structure in controlled aging experiments can be determined by differential scanning calorimetry
[189]	Odegard and Bandyopadhyay	Physical aging of epoxy polymers and their composites	Literature review	The physically aged epoxy polymers lost weight due to the deprivation of residual volatiles as well as moisture.	The physical properties of epoxy polymers are affected by mechanisms that can undermine their reliability in terms of engineering components as well as structure
